# Development of high affinity antibodies to *Plasmodium falciparum* merozoite and sporozoite antigens during infancy and adulthood

**DOI:** 10.3389/fimmu.2025.1562671

**Published:** 2025-07-02

**Authors:** Allan Lugaajju, Richard Idro, Stephen Kiwuwa, James G. Beeson, Damien R. Drew, Susanne E. Mortazavi, Sara Linse, Kristina E. M. Persson

**Affiliations:** ^1^ College of Health Sciences, Makerere University, Kampala, Uganda; ^2^ Burnet Institute, Melbourne, VIC, Australia; ^3^ Department of Infectious Diseases, University of Melbourne, Melbourne, VIC, Australia; ^4^ Department of Microbiology and School of Translational Medicine, Monash University, Melbourne, VIC, Australia; ^5^ Department of Laboratory Medicine, Lund University, Lund, Sweden; ^6^ Department of Infectious Diseases, Skåne University Hospital, Lund, Sweden; ^7^ Department of Biochemistry and Structural Biology, Lund University, Lund, Sweden; ^8^ Clinical Chemistry and Pharmacology, Laboratory Medicine, Skåne University Hospital, Lund, Sweden

**Keywords:** malaria, *falciparum*, affinity, antibodies, CSP, AMA1, MSP2

## Abstract

Antibodies are important for protection against malaria. For optimal protective activity, it is thought that antibodies need to have high affinity. A longitudinal study conducted in Uganda followed newborn infants and their mothers for nine months. The study found that antibody affinity (here measured as dissociation rate constant, k_d_) against the merozoite antigens AMA1 and MSP2 decreased from birth to six months in the infants, then gradually increased to 9 months, but not reaching the level observed in the mothers. In contrast, affinity against the sporozoite antigen CSP, did not change throughout the study period. Among mothers, no significant changes in antibody affinity were observed for any antigen, which is consistent with expectations for adults in an endemic area. Comparing specific antibody affinities to total antibody levels revealed almost no correlations, indicating that antibody magnitude and affinity evolve differently during immune development. Significant correlations were observed between antibody affinities and some atypical memory B cells. In conclusion, our study shows that development of naturally acquired slowly dissociating (high affinity) antibodies against malaria can evolve separately across different antigens. This is important information for future vaccine development studies.

## Introduction

Malaria remains a major global health challenge, causing significant morbidity and mortality. In 2023, an estimated 263 million malaria cases and 597,000 deaths were reported globally (1). Despite progress in reducing malaria incidence and mortality, challenges such as drug resistance, limited vaccine efficacy, and healthcare access persist, underscoring the need for continued innovations and investments (1).

Naturally-acquired immunity against malaria develops slowly and typically requires repeated exposure (2). While the precise mechanisms underlying the immunological responses are not fully understood, antibodies play a crucial role in mitigating clinical *P. falciparum* malaria. Studies as early as the 1960s demonstrated reduced parasitemia and clinical symptoms following the passive transfer of immunoglobulins from immune donors to infected individuals (3, 4). Antibodies targeting merozoite proteins, such as Merozoite Surface Protein 2 (MSP2) and Apical Membrane Antigen 1 (AMA1), or surface antigens on infected red blood cells, are integral to acquired immunity against malaria and are potential antigen candidates for vaccine development (5–10).

MSP2 is a 25–30 kDa protein (11) abundantly expressed on the surface of merozoites, playing a key role in red blood cell invasion (12). Both vaccine-induced and naturally acquired antibodies targeting MSP2 are strain-specific and protective (13, 14). These antibodies can mediate complement fixation to inhibit invasion (15) and promote opsonic phagocytosis (16). Similarly, AMA1 (82 kDa) is expressed on both sporozoites and on merozoites (17), with antibodies against it impacting the invasion of hepatocytes and erythrocytes (18, 19). The circumsporozoite protein (CSP) is the most abundant protein on the surface of the *Plasmodium* sporozoites and serves as the antigenic target of the RTS,S and R21 vaccines, which are recommended by WHO for implementation (1). However, the long-term efficacy of these vaccines still requires improvement (20–31). Antibodies to CSP can promote complement fixation, enhance opsonic phagocytosis and inhibit invasion by sporozoites (32–34).

Developing more efficacious vaccines requires a deeper understanding of the factors limiting their current efficacy. One significant challenge is the lack of an *in vitro* immunologic correlate of protection against malaria infection and disease (35). Understanding the development of both humoral and cellular immunity is crucial for designing effective malaria vaccines. Mere presence of antibodies does not suffice for immunity against malaria; the quality of the antibodies is equally essential (36). High-affinity antibodies, produced through effective priming by antigens or vaccines, mark the maturation of specific B cell clones (37). The generation of these high-affinity antibodies depends on repeated malaria exposure and clinical manifestations of the disease (38, 39).

Various methods have been used to evaluate antibody affinity. For example, an urea-based Enzyme-Linked Immunosorbent Assay (ELISA) has shown that individuals with complicated malaria has lower-affinity antibodies compared to those with asymptomatic or uncomplicated malaria (39). High-affinity antibodies targeting the parasite antigen VAR2CSA have been associated with reduced placental malaria (40). On the contrary, other studies have found no correlation between the affinity of antibodies against specific blood-stage malaria antigens and clinical protection (41–43). Of recent, high-affinity antibodies against the CSP N-terminal domain were found to fail in inhibiting *P. falciparum* activity (44).

These inconsistencies may be attributed to inadequacies in the methods used, or structural variations in the antigens studied (38). In previous work, we demonstrated that ELISA-based avidity assays strongly correlated with standard ELISA results but provided limited additional information for our antigens. This prompted us to explore alternative methods (45). Surface Plasmon Resonance (SPR) has been recommended for assessing vaccine efficacy in various diseases (46) and has shown promise in malaria research, demonstrating correlations between high-affinity antibodies and both malaria protection (45) and with invasion-inhibitory functions (38).

While numerous studies have explored antibody affinity against potential malaria antigen candidates for vaccine development, few have investigated how antibody affinity develops over extended periods in early infancy, particularly in comparison to maternal antibodies, This study aimed to evaluate the dissociation rate constant, k_d_ (which correlates with affinity) of naturally acquired antibodies targeting key immune and vaccine antigens - AMA1, MSP2 and CSP. We conducted a longitudinal study in Uganda, analyzing samples collected from infants at birth and at 2.5, 6, and 9 months of age using SPR. Additionally, we compared antibody dissociation rate constants between mothers and infants, correlating the results with total antibody levels and the presence of different B cell subsets.

## Materials and methods

### Samples

Samples from 68 mother-infant pairs were randomly selected from a previously described cohort (47). Briefly, plasma samples were collected from mothers and babies in a malaria endemic area 20 km northeast of Kampala in Uganda, at the antenatal center at Kasangati Health Centre. In brief, blood was collected in lithium heparin vacutainer tubes and plasma was separated after centrifugation at room temperature, then the plasma samples were stored at -80°C. In this study area, malaria is meso-endemic with peak transmission after the two rainy seasons (February–March and September–October) every year, with an estimated malaria prevalence of 13% in children (48). Eligibility criteria were normal deliveries with healthy newborns and agreement to attend follow-up visits at 2.5, 6 and 9 months of the child’s age. Each pregnant woman received at least one dose of intermittent preventive treatment and was provided with a long-lasting insecticide-treated bed net. Detailed clinical examinations were conducted at recruitment and during follow-up visits, with data entered into the study questionnaire. Malaria rapid diagnostic tests (RDTs) were performed on all samples, and blood smear examinations were done upon a positive RDT. None of the individuals exhibited signs of severe infection during recruitment or sampling.

### Surface plasmon resonance

The SPR dissociation rate constant measurements were performed using a Biacore 3000 instrument, (GE Healthcare Life sciences, Uppsala) as described before (45). Briefly, CM5 sensor chips (Pharmacia biosensor AB, Uppsala Sweden) were activated by using an amine coupling kit (GE Healthcare Bio-Sciences AB, Uppsala Sweden) using an injection pulse (10 min, 5 µL/min). The AMA1, MSP2 and CSP recombinant proteins were immobilized using a manual injection of 100 µg/mL in coating buffer (0.01 M sodium acetate buffer, pH 4.0) until the desired response units were achieved. The unoccupied activated sites were blocked by ethanolamine. All steps were carried out in a continuous flow of HBS-EP (10 mM Hepes, 150 mM NaCl, 3 mM EDTA, 0.005% polysorbate 20) running buffer at 5 µL/min. The residual antibodies that could remain attached to the immobilized antigens after measurement of k_d_ were removed by washing with 10 mM glycine-HCl (pH 1.5) for 5 seconds at 5 µL/min to regenerate the surface before injection of the next plasma sample. An internal control, which was a pool of Ugandan adult (presumably immune) samples, was injected after a complete set of mother-baby pair samples (equal to 6 plasma samples) to test the stability of the immobilized protein and the reproducibility of the assay. The background level was determined by Swedish non-immune plasma and all the samples considered in the subsequent analysis were above the background level. The response was monitored as a function of time (sensogram) at 25 °C. The intra-assay coefficient of variation (CV), based on repeat measurements of the same sample within a single run, varied between 4-6% for the different antigens. The inter-assay CV, calculated from measurements across different days and chips, was below 5% for all three antigens. The BIAevaluation 4.1 software was used to fit a single exponential decay to the data to estimate the k_d_ values. AMA1 and CSP were expressed in HEK293 cells with a his-tag and purified using nickel columns (49–51). MSP2 was expressed in *E. coli* and purified as described (52).

### ELISA

ELISA was performed as described before, using schizont extract from *P. falciparum* cultures (47). In brief, microtiter plates were prepared by coating with schizont extract, blocking with 5% skimmed milk (Sigma) for the IgG assay and super block dry blend (Thermo Scientific) for the IgM assay. Plates were then incubated with peroxidase-conjugated goat anti-human IgG or IgM antibodies (Sigma). Antibody binding was detected using TMB (3,3′,5,5′-Tetramethylbenzidine) substrate (Promega), and the optical density (OD) was measured at 450 nm.

### Flow cytometry of CD19+ B-cells

Studies of different populations of total and *P. falciparum*-positive B-cells was performed as described before (47). Subpopulations of cells were determined: total/Pf+ IgG memory B-cells (MBC) (IgG^+^ MBC) [CD19^+^CD20^+^CD27^+^IgG^+^), non-IgG+ MBC (CD19^+^CD20^+^CD27^+^IgG^−^), atypical MBC (CD19^+^CD20^+^CD27^−^IgG^+^), naïve B-cells (CD19^+^CD20^+^CD27^−^IgG^−^) and plasma cells/blasts (CD19^+^CD20^−^CD27^+^IgG^−^).

### Statistical analysis

Continuous variables were presented as medians with interquartile ranges or estimated means with corresponding confidence intervals. To account for the repeated measurements, linear mixed models were employed to model how each protein changed over time with a first-order autoregressive covariance structure, considering individuals as a random effect. Correlations between protein levels at each time point were analyzed using Spearman’s correlation for each time point, separately for mothers and infants. False discovery rate adjustments were applied to correct for multiple comparisons. Antibody dissociation rate constants between groups were compared using non-parametric Mann-Whitney U Test. For multiple group and antigen comparisons, Kruskal-Wallis was employed. Two-sided p-values were calculated for all tests, with p < 0.05 considered statistically significant. All analyses were performed using R (v. 4.1.2) or GraphPad Prism, version 10 (GraphPad Software Inc., San Diego, CA, USA).

## Results

### Estimating antibody affinity through dissociation rate constant measurements

We used SPR to quantify the dissociation rate constant (k_d_) of antibody binding to immobilized antigens. We have previously evaluated this method for estimating affinity differences of polyclonal antibodies in human serum and confirmed the reproducibility of the method (45, 53). Three targets of acquired immunity and potential malaria antigen candidates for vaccine development, including two merozoite antigens (AMA1, MSP2) and one sporozoite protein (CSP), were immobilized through amine coupling to SPR senor chips. The dissociation rate constant (k_d_) for the complexes between antibody and these antigens in the individual mother-baby pair plasma samples was used to estimate the affinity. Plasma dilutions of 1:15 and 1:30 were used and it was found that the k_d_ values were independent of the plasma concentration, as expected. A pool of plasma samples from a malaria endemic area was used regularly as an internal control to test the stability of the antigens on the sensor chip, and it was found that the k_d_ values were not dependent on protein loss over time.

### Total anti *P. falciparum* IgG and IgM

Antibody levels against *P. falciparum* were analyzed in all participants, as previously reported (47, 54). In this study, we re-evaluated the data using linear mixed models to assess the development of total anti-*P. falciparum* IgG and IgM in infants. As expected, infants exhibited high levels of anti-*P. falciparum* IgG levels at birth, which declined by 2.5 months and then stabilized over the next 9 months after birth. In contrast, anti-*P. falciparum* IgM levels increased gradually from 6 months onwards, reflecting the infants’ subsequent exposure to malaria. Among mothers, no significant differences in anti-*P. falciparum* IgG or IgM levels were observed between the two time points.

### Development of high affinity antibodies during infancy

**Figure 1:**
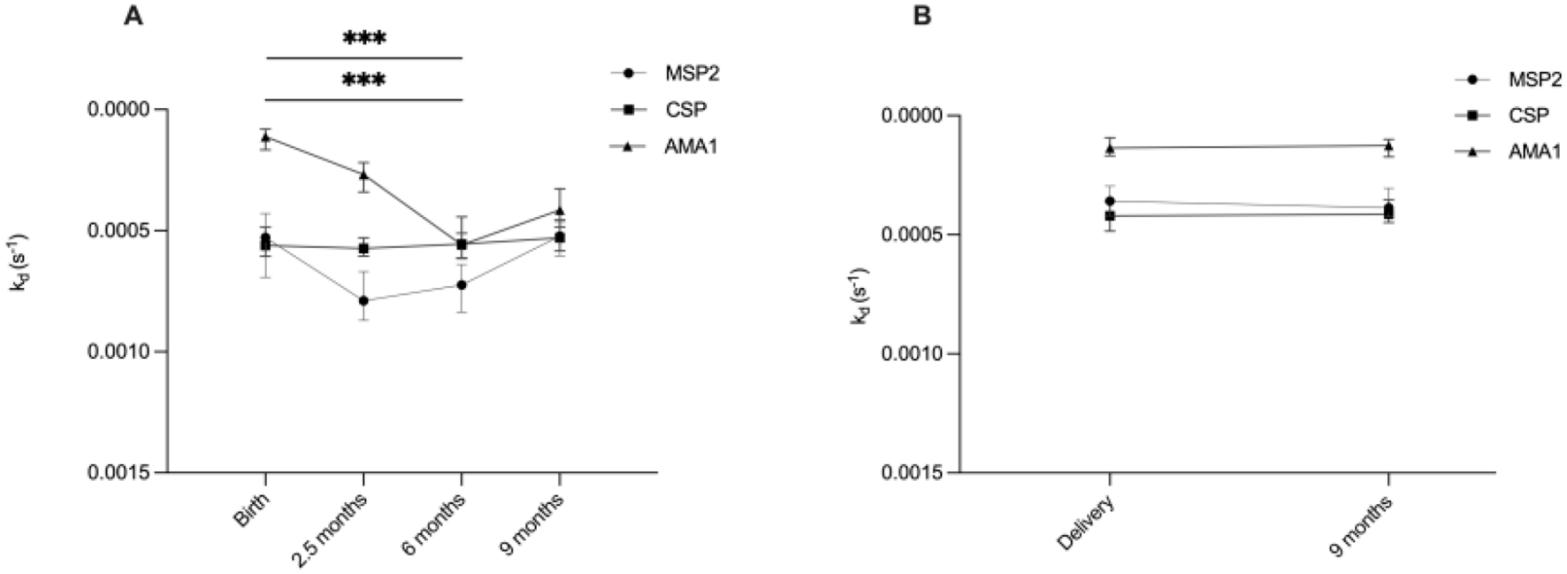
Overview of development of high affinity antibodies during infancy and in mothers. Graphs illustrate estimated means of antibody dissociation rate constants (k_d_) for MSP2, CSP and AMA1 during **(A)** infancy (at birth, 2.5, 6 and 9 months) and **(B)** in mothers (at delivery and after 9 months). Lower k_d_ values indicate higher antibody affinity. *** indicates significant at P < 0.001, analyzed by linear mixed models.

We conducted a linear mixed model to assess the changes in antibody affinities against specific malaria antigens in infants at various time points from birth until 9 months of age. The estimated means of dissociation rate constants for antibodies targeting AMA1 and MSP2 significantly increased from birth to six months, then decreased by nine months (**Figure 1A**). However, the estimated means of antibody dissociation rate constants for CSP did not show significant changes over the 9 months (**Figure 1A**).

### Stable affinity in the mothers

The estimated means of antibody dissociation rate constants in the mothers for the antigens AMA1, MSP2 and CSP were the same at delivery and nine months postpartum (**Figure 1B**).

### Higher antibody affinity in mothers compared to infants

**Figure 2:**
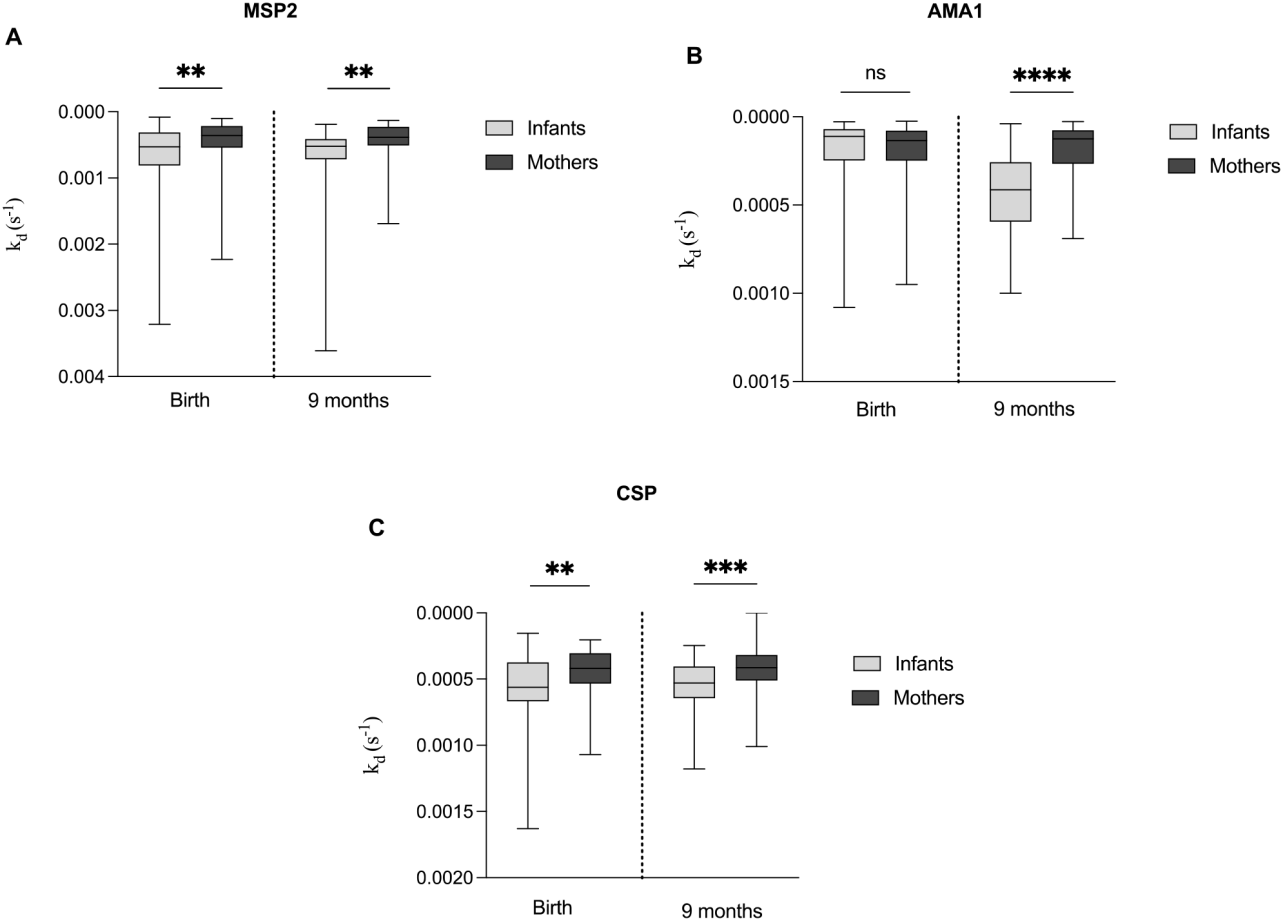
Comparative analysis of antibody dissociation rate constant **(k_d_)** for MSP2, AMA1 and CSP in infants and mothers. Box plots comparing the median k_d_ of antibodies targeting the antigens **(A)** MSP2, **(B)** AMA1 and **(C)** CSP between mothers at delivery and nine months postpartum, and their infants at birth and nine months. Box plots represent interquartile range (IQR), whiskers the range and horizontal lines the median; **, ***, **** indicate significant differences (p < 0.01, 0.001, < 0.0001, respectively), and ns (p ≥ 0.05) indicates not significant tested by the Mann-Whitney U test.

We compared the antibody dissociation rate constants of mothers at delivery and nine months postpartum with those of their infants at the same time points. At both time points, mothers exhibited significantly lower median of k_d_ (indicating higher affinity) for all three antigens AMA1, MSP2, and CSP compared to their infants, except for AMA1 at delivery, where no significant difference was observed (**Figures 2A–C**).

### Difference in antibody affinity between antigens

**Figure 3:**
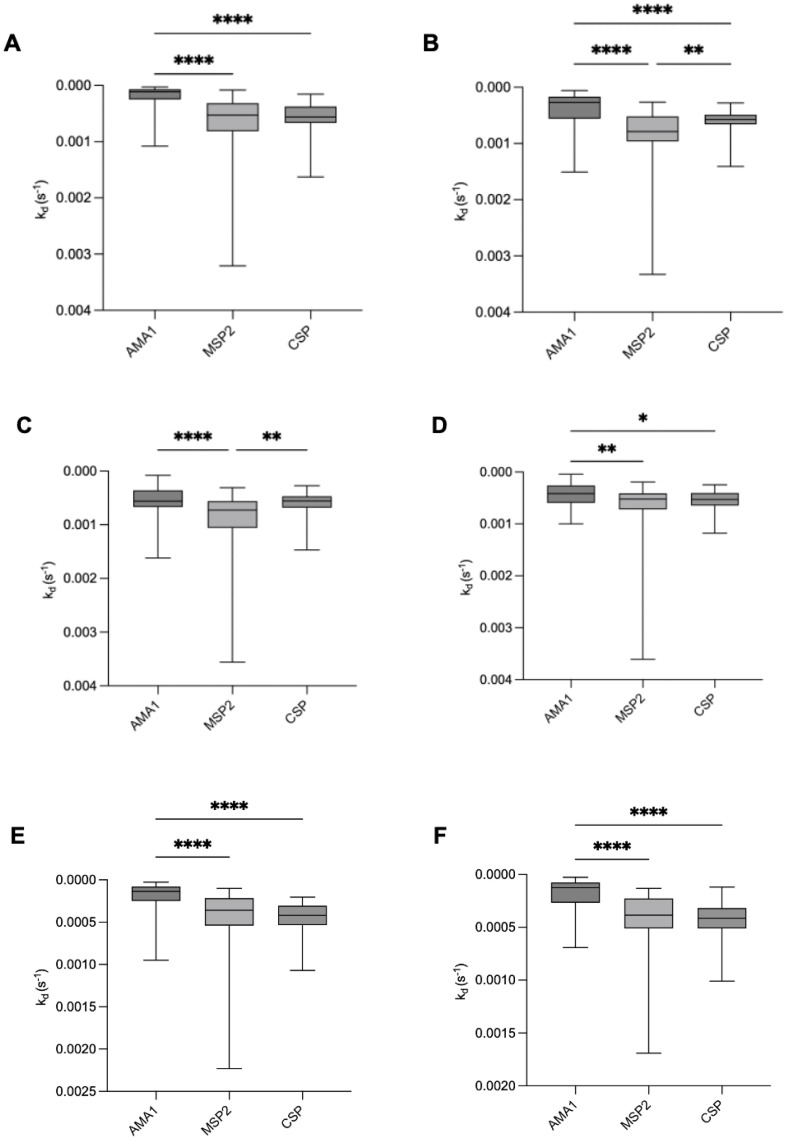
Comparison of antibody dissociation rate constants for AMA1, MSP2, and CSP in infants and mothers. Box plots illustrate the median antibody affinities (k_d_) against AMA1, MSP2, and CSP in infants at **(A)** birth, **(B)** 2.5 months, **(C)** 6 months, **(D)** 9 months, and in mothers at **(E)** delivery and **(F)** 9 months postpartum. Box plots represent interquartile range (IQR), whiskers the range, and horizontal lines represent the median; *,**, ***, and **** indicate significant differences (p < 0.05, 0.01, 0.001, and 0.0001, respectively) tested by Kruskal-Wallis test.

To investigate differences in antibody dissociation rate constants among the three tested antigens, we compared median dissociation rate constants to AMA1, MSP2, and CSP in both infants and mothers throughout the study period (**Figures 3A–F**). At birth and up to 2.5 months, infants exhibited significantly slower dissociation for AMA1 compared to MSP2 and CSP. By 6 and 9 months of age, no significant difference was observed between the dissociation rate constants for AMA1 and CSP, while MSP2 showed the highest dissociation rate constant (lowest affinity). For mothers, both at delivery and nine months postpartum, we observed similar patterns as for the infants, with significantly lower median k_d_ values for AMA1 compared to MSP2 and CSP (**Figures 3E, F**).

### Relationship between antibody affinity and total levels of anti-*P. falciparum* IgG and IgM

**Figure 4:**
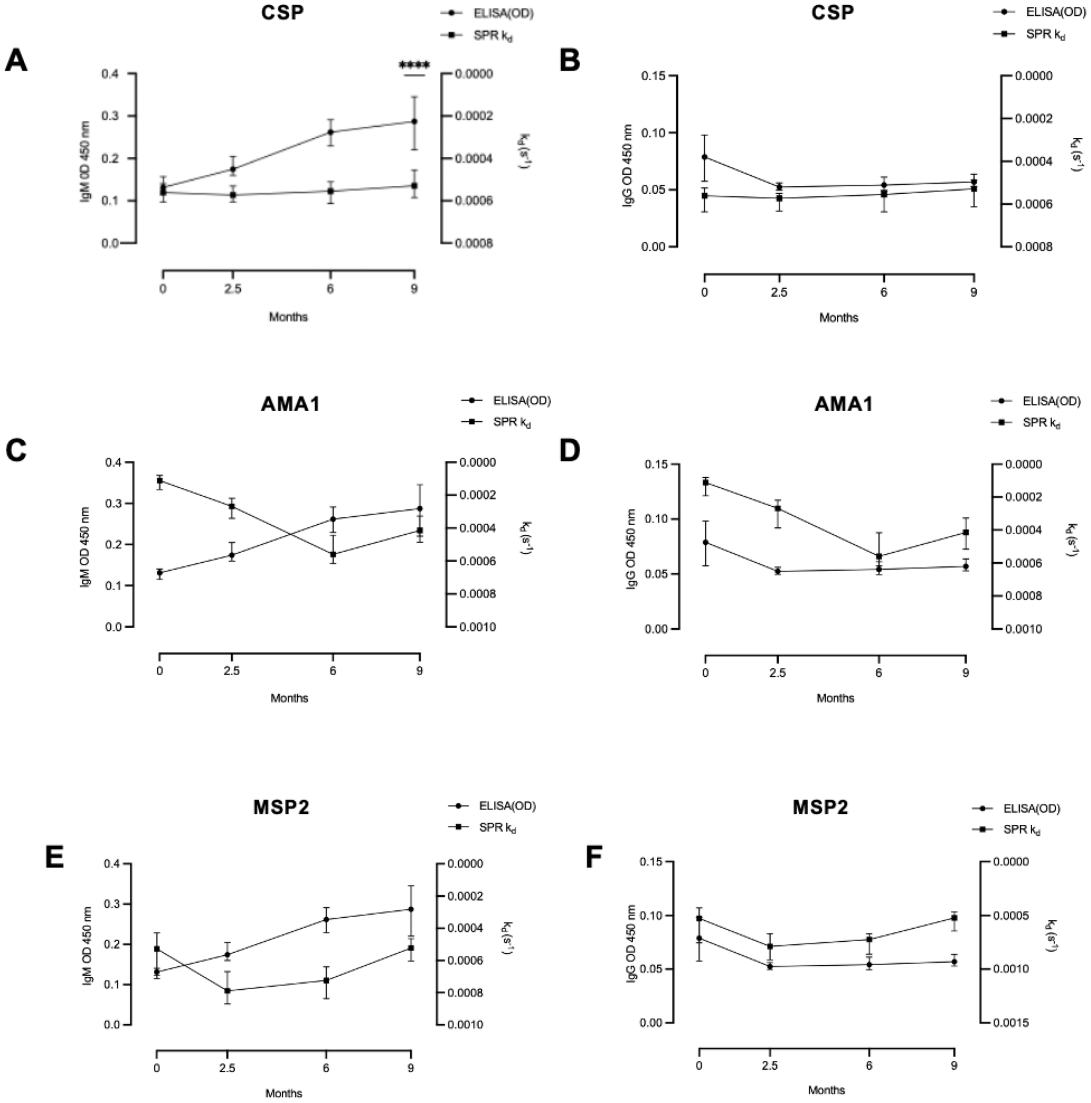
Relationship between antibody dissociation rate constant and ELISA-determined antibody levels. In infants there was **(A)** a significant correlation between antibody level and dissociation rate constant only for IgM and CSP at 9 months, but not for any other tested correlations: **(B)** IgG and CSP, **(C)** IgM and AMA1, **(D)** IgG and AMA1, **(E)** IgM and MSP2 and **(F)** IgG and MSP2. Coefficient of correlation, rho, and P values were calculated using Spearman’s correlation test. **** significant at P < 0.0001.

We compared the antibody dissociation rate constant for the tested antigens with total levels of *P. falciparum* IgG and IgM (**Figure 4**). No significant correlations were observed between antibody dissociation rate constants and total IgG or IgM at any time point, except for a negative correlation between CSP dissociation rate constant and *P. falciparum* IgM in infants at 9 months of age (rho= -0.47, p < 0.0001).

### Correlation between antibody affinity and total CD19-positive B cell and various *P. falciparum* specific B cells

**Figure 5:**
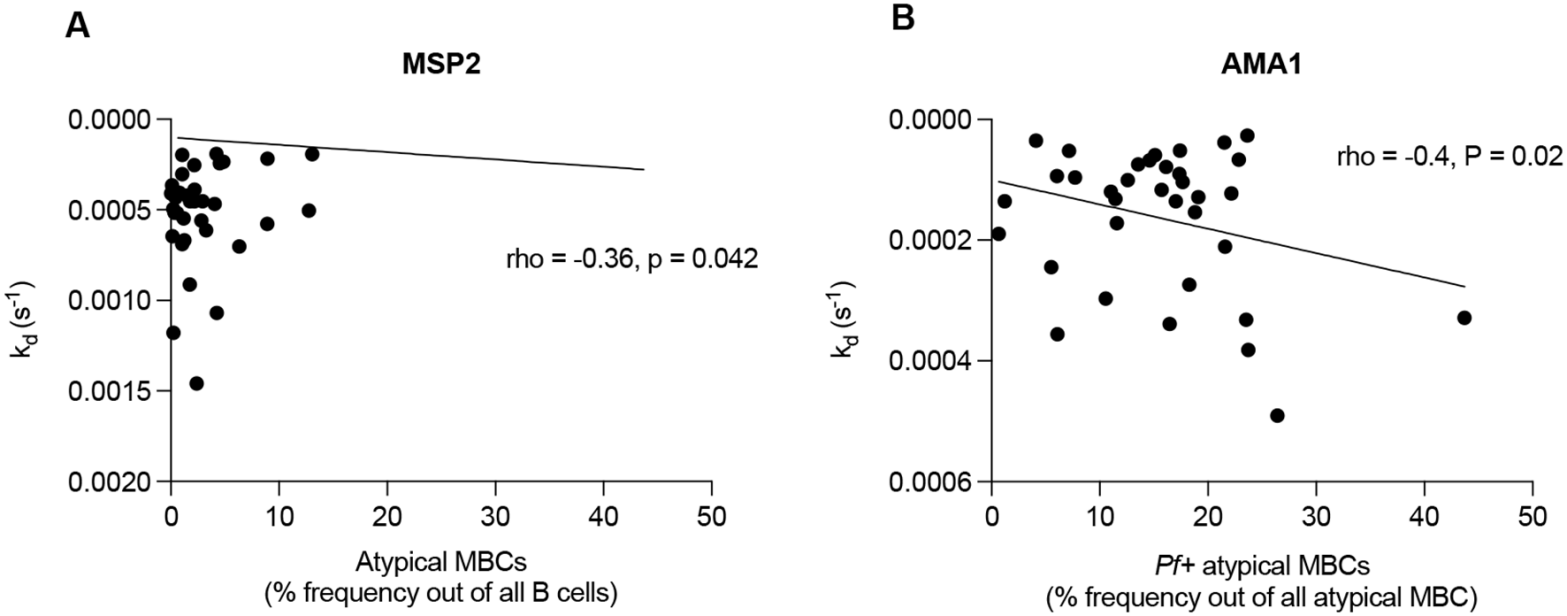
Correlations between antibody affinity and B cell subsets. Figures representing **(A)** antibody dissociation rate constant for MSP2 and atypical memory B cells (MBCs) in infants at 9 months and **(B)** antibody dissociation rate constant for AMA1 and *Pf+* atypical MBCs in mothers postpartum. Pearson’s coefficient was used to detect correlations between plasma antibody affinity and B cell subsets.

We previously quantified levels of total CD19-positive B cells and *P. falciparum* specific (*Pf+*) B cell subsets in this cohort (47). In this study we performed a statistical reanalysis to investigate correlations between antibody dissociation rate constant and various *Pf+* B cell subsets, namely, *Pf+* IgG MBC, *Pf+* non-IgG^+^ MBC, *Pf+* atypical MBC, *Pf+* naïve B-cells and *Pf+* plasma cells/blasts. Our analysis revealed correlations between antibody dissociation rate constant for MSP2 and atypical memory B cells (MBCs) in infants at 9 months (rho = -0.36, p = 0.042) (**Figure 5A**). Additionally, in mothers 9 months postpartum, there were correlations between AMA1 and non-IgG MBCs (rho = 0.39, p = 0.024), as well as between AMA1 and *Pf+* atypical MBCs (rho = -0.4, p = 0.02 (**Figure 5B**).

## Discussion

In this study, we investigated the development of antibody affinity against three antigen candidates for vaccine development: MSP2, AMA1 and CSP through measurements of dissociation rate constants. Our results show that antibodies targeting AMA1 exhibited the slowest dissociation, followed by CSP and then MSP2. This finding aligns with previous research indicating that antibodies from residents in malaria-endemic areas tend to develop higher affinity antibodies to AMA1 compared to MSP2 (45, 55). In vaccine trials, it has also been shown that maintaining high affinity antibodies against MSP2 over time can be challenging (53). Moreover, the k_d_ values for AMA1 observed in our study were comparable to those of monoclonal antibodies generated from B cells of a donor living in an endemic area (56). The slower dissociation observed for AMA1 may be due to its structural stability, which is attributed to multiple intramolecular disulphide bonds linking its globular domains (57, 58).

In contrast, MSP2, is considered an unstructured protein, which likely contributes to its lower stability. MSP2 has been evaluated in a vaccine trial called Combination B, which included recombinant *P. falciparum* ring-infected erythrocyte surface antigen alongside MSP1 and MSP2. This trial demonstrated a reduction in parasite density and showed that the vaccine exerted selective pressure on infecting *P. falciparum* strains (59). A phase 1 trial for the MSP2-C1 vaccine, containing recombinant forms of both *msp2* alleles families (3D7 and FC27), found that MSP2-specific antibodies did not directly inhibit parasite growth *in vitro.* Instead, these antibodies acted via antibody-dependent cellular inhibition (ADCI) to inhibit parasite growth (60), as well as activated complement on the merozoite surface and promoted opsonic phagocytosis (15, 16). Similarly, human anti-AMA1 antibodies have been shown to inhibit *P. falciparum in vitro* (56). A field trial using the FMP2.1/AS02 (A) vaccine, which contains AMA1 from the 3D7 strain of *P. falciparum*, did not provide significant protection against clinical malaria but exhibited evidence of strain-specific efficacy (61). These findings suggest that AMA1 could be a valuable component of a multi-component vaccine. We did not observe strong correlations between total levels of anti-*P. falciparum* antibodies and the specific dissociation rate constants measured. This lack of correlation may reflect insufficient levels of individual specific antibodies, or that the antibody levels evolved differently compared to antibody affinities.

Our study aimed to assess the development of antibody affinity during infancy and compare it with that of mothers. We measured the dissociation rate constant (k_d_) of antibody binding to immobilized antigens because k_d_ is independent of concentration and reliable values are more easily accessible than affinity, which requires precise knowledge of plasma concentrations for accurate quantification. Moreover, the lifetime of the antigen-antibody complex, i.e. the inverse of the dissociation rate constant, may be the determinant of function. In infants, we observed a significant increase in antibody dissociation rate from AMA1 and MSP2 from birth to six months, likely due to the gradual waning of maternal IgG antibodies transferred transplacentally (62, 63). This aligns with previous findings showing that maternal antibodies against malaria typically wane within the first 3 to 6 months of life (64–66). After six months, antibody dissociation rate constants to AMA1 and MSP2 decreased, probably as a result of exposure to malaria parasites in this endemic area. Concurrently, overall antibody levels also increased. Previous studies have demonstrated that malaria exposure enhances antibody affinities to AMA1 and MSP2 (38, 39), in line with the exposure-dependent nature of malaria immunity (14, 67).

In mothers, antibody dissociation rates from AMA1 and MSP2 were lower than those observed in infants and were the same at delivery and nine months postpartum. This stability reflects the well-documented phenomenon in endemic regions where repeated malaria exposures lead to a plateau in antibody affinity among adults. Interestingly, significant differences in antibody dissociation rate constants between mothers and infants at birth were observed for MSP2 and CSP, with a non-significant trend for AMA1. Mothers exhibited lower dissociation rate constants (higher affinities), suggesting that transplacental transfer does not preferentially transfer the highest affinity antibodies. This difference may be explained by the fact that infants only receive IgG through transplacental transfer, whereas maternal samples contain both IgG and IgM. Anti-malarial IgM could exhibit higher affinity than IgG (68), this difference may reflect the absence of IgM in infants.

Previous studies have shown that high-avidity antibodies for antigens such as tetanus toxoid and type 3 pneumococcal antigen are preferentially transferred through the placenta. This selectivity may depend on the antigen rather than the degree of antigenic exposure (69). Similarly, a study on pertussis toxin antibodies confirmed preferential placental transfer of high-avidity antibodies but noted decreased transfer in HIV-positive mothers (70). In our cohort, none of the mothers were HIV-positive. Additionally, the transplacental transfer of IgG3 has been associated with a reduced risk of clinical malaria (71, 72). However, preferential transfer generally follows the order: IgG1 >IgG4 >IgG3 >IgG2 (73). This raises the possibility that high-affinity antibodies for MSP2 and CSP may belong to subclasses that are less efficiently transferred. Selective transfer mechanisms or other unknown factors may influence the quality and subclass distribution of antibodies passed from mother to infant. To help elucidate the affinity and functional relevance of antibodies, IgG subclass-specific affinity assays could be performed, which would include functional assays such as opsonic phagocytosis and parasite growth inhibition to link affinity maturation to protective efficacy. Previous studies have shown that high-affinity antibodies against AMA1 and MSP2 correlate with greater opsonic and inhibitory activity (15, 74). Even though we did not include such assays in this study, our findings provide a basis for future research to investigate whether the observed affinity profiles, particularly for AMA1 and MSP2, translate into functional protection.

In comparison to the observed changes in antibody dissociation rate constants for AMA1 and MSP2 in infants during the 9 months study, no significant changes in antibody dissociation rate constant for CSP were observed, either in infants or in adults. Previous research has shown that most natural antibody responses to CSP target the central NANP repeat region (28, 75–77), which is intrinsically disordered (78) and tends to elicit low affinity antibodies (79), as well as the C-terminal region (80). Moreover, earlier studies have highlighted challenges in maintaining a robust response for CSP over time and in achieving high efficacy (81–83). We speculate that the difficulty in inducing high-affinity antibodies may contribute to CSP’s modest success as a vaccine candidate. Even in mothers, antibodies against CSP showed higher dissociation rate constant (lower affinity) compared to AMA1, although they were similar to those against MSP2. This suggests that the structural characteristics of CSP may be of importance when selecting and combining antigens for optimal vaccine design.

Despite variations in antibody affinities for different antigens, the responses in mothers remained the same at the time of delivery and nine months later for all tested antigens. This implies that in adults, each of the three antigens elicits an immunological response from germinal center B cells that have undergone both somatic hypermutation and clonal selection (84, 85). These activated B cells appear to reach a threshold level of antigen-specific affinity maturation, beyond which no further increase in affinity is observed (38). This phenomenon has also been observed in other diseases, such as vesicular stomatitis virus (86), and in vaccination against meningococcus using a recombinant vaccine, particularly after the third and fourth doses (87).

It has been shown that both classical and atypical memory B cells generated following natural *P. falciparum* infection produce neutralizing antibodies against blood stage *P. falciparum* parasites. However, only atypical memory B cells appear to show evidence for active antibody secretion (88). These atypical memory B cells are largely maintained by chronic malaria exposure (89–91), with subsets such as IgD^+^IgM^lo^ and IgD^-^IgG^+^ displaying a high threshold for antigen avidity during activation. This mechanism likely helps prevent autoimmune responses during chronic malaria (92)., highlighting the important role of these cells in the development of malaria immunity (47, 93). Furthermore, we identified positive correlations between atypical memory B cells and antibody affinities against the tested parasite antigens, emphasizing their significance in malaria immunity. This finding is worth considering in future vaccine designs aimed at improving efficacy, especially given the modest efficacy observed with current vaccines like RTS, S and R21.

In conclusion, our study demonstrates a distinct and gradual development of antibody affinity for the merozoite antigens AMA1 and MSP2 during the first nine months of life, but not for the sporozoite antigen CSP. In adults, antibody affinity was relatively the same at delivery and nine months post-partum. Additionally, we identified correlations between antibody dissociation rate constant and atypical memory B cells. These findings contribute to our understanding of naturally acquired immunity to malaria and provide valuable insights for selecting optimal antigen candidates for vaccine development in future clinical trials.

## Data Availability

The original contributions presented in the study are included in the article/**Supplementary Material**. Further inquiries can be directed to the corresponding author.
