# Supplementary material for: Development of high affinity antibodies to Plasmodium falciparum merozoite and sporozoite antigens during infancy and adulthood
Source: Front Immunol. 2025 Jul 2;16:1562671. doi: 10.3389/fimmu.2025.1562671 (PMC12263619; doi:10.3389/fimmu.2025.1562671)
Supplement: Supplementary file 1 [file DataSheet1.pdf]

Mother baby pair affinity (kd) data for AMA1, MSP2 and CSP

SNO	ID	Mo			B0			B2.5
		AMA1	MSP2	CSP	AMA1	MSP2	CSP	AMA1
1	5	2,72E-04	4,23E-04	4,92E-04	1,86E-04	5,78E-04	1,04E-03	4,59E-04
2	6	3,81E-04	1,00E-04	3,60E-04	3,93E-04	1,21E-04	5,48E-04	6,94E-04
3	8	7,24E-05	3,60E-04	5,06E-04	6,67E-05	4,14E-04	5,65E-04	7,64E-05
4	9	9,91E-05	1,88E-04	3,05E-04	4,60E-05	8,06E-04	4,87E-04	1,25E-04
5	10	8,03E-05	3,61E-04	5,36E-04	7,32E-05	4,30E-04	5,76E-04	3,53E-04
6	11	9,42E-05	1,15E-04	2,25E-04	8,44E-05	2,34E-04	1,77E-04	2,42E-04
7	12	7,42E-05	1,04E-04	3,39E-04	1,07E-04	3,64E-04	4,34E-04	2,70E-04
8	13	8,09E-05	1,02E-04	3,40E-04	8,22E-05	8,92E-05	3,12E-04	8,98E-04
9	15	3,36E-04	5,62E-04	4,52E-04	3,76E-04	8,52E-04	5,62E-04	7,56E-04
10	16	1,14E-04	2,86E-04	3,67E-04	5,15E-05	3,17E-04	5,45E-04	1,64E-04
11	18	5,62E-05	3,12E-04	3,72E-04	4,63E-05	4,38E-04	5,56E-04	1,48E-04
12	19	6,13E-05	1,37E-04	2,46E-04	4,40E-05	1,14E-04	4,00E-04	6,03E-05
13	20	1,01E-04	4,10E-04	4,07E-04	9,85E-05	4,63E-04	3,86E-04	2,17E-04
14	21	2,53E-05	3,20E-04	2,98E-04	5,06E-05	5,82E-04	3,63E-04	1,72E-04
15	23	1,38E-04	4,69E-04	6,46E-04	1,93E-04	8,97E-04	5,68E-04	3,06E-04
16	24	7,70E-04	8,01E-04	8,29E-04	1,08E-03	1,29E-03	1,63E-03	1,51E-03
17	26	9,50E-04	2,23E-03	5,25E-04	8,42E-04	2,65E-03	5,63E-04	3,42E-04
18	27	2,42E-04	9,18E-04	1,07E-03	5,89E-04	3,21E-03	8,60E-04	9,60E-04
19	30	2,35E-04	2,06E-03	9,01E-04	6,36E-04	3,20E-03	8,06E-04	9,52E-04
20	32	6,69E-05	5,95E-04	2,21E-04	4,88E-05	9,44E-04	1,54E-04	5,17E-04
21	34	1,23E-04	4,74E-04	7,58E-04	1,06E-04	5,77E-04	7,85E-04	3,92E-04
22	36	2,53E-04	3,19E-04	2,47E-04	2,05E-04	5,56E-04	3,20E-04	6,25E-04
23	37	1,62E-04	6,21E-04	2,91E-04	8,39E-05	7,04E-04	2,37E-04	2,68E-04
24	38	2,57E-04	4,24E-04	4,28E-04	2,19E-04	5,21E-04	4,65E-04	2,84E-04
25	39	8,77E-05	4,13E-04	3,48E-04	7,13E-05	1,09E-03	6,37E-04	2,64E-04
26	41	7,85E-05	6,40E-04	2,80E-04	1,58E-04	8,05E-04	3,80E-04	4,70E-04
27	42	9,03E-05	1,29E-04	4,24E-04	7,84E-05	1,47E-04	3,73E-04	1,04E-04
28	43	2,35E-04	4,21E-04	4,17E-04	2,10E-04	5,15E-04	5,35E-04	5,94E-04
29	48	2,90E-04	2,70E-04	3,58E-04	3,39E-04	3,05E-04	3,57E-04	6,89E-04
30	49	1,57E-04	1,30E-04	2,95E-04	1,17E-04	1,32E-04	2,75E-04	3,17E-04
31	50	5,91E-05	3,00E-04	4,50E-04	4,71E-05	4,32E-04	5,38E-04	1,10E-04
32	55	1,99E-04	5,18E-04	2,96E-04	1,71E-04	8,71E-04	3,61E-04	4,66E-04
33	56	4,48E-05	3,24E-04	2,77E-04	2,87E-05	4,16E-04	3,65E-04	1,27E-04
34	57	6,92E-05	2,73E-04	2,39E-04	5,34E-05	3,55E-04	8,21E-04	7,93E-05
35	58	9,92E-05	5,49E-04	4,66E-04	5,42E-05	1,33E-03	7,13E-04	1,95E-04
36	59	7,96E-05	1,87E-04	2,12E-04	7,31E-05	2,50E-04	5,52E-04	5,96E-05
37	66	1,67E-04	1,65E-04	3,78E-04	1,56E-04	1,95E-04	5,60E-04	2,52E-04
38	67	1,33E-04	2,14E-04	2,58E-04	9,59E-05	7,57E-04	3,10E-04	2,37E-04
39	68	3,46E-04	4,81E-04	5,69E-04	3,27E-04	6,69E-04	6,72E-04	6,31E-04
40	69	1,69E-04	2,44E-04	3,57E-04	1,42E-04	2,57E-04	3,45E-04	4,40E-04
41	71	3,69E-05	4,11E-04	4,03E-04	4,69E-05	5,13E-04	3,82E-04	1,88E-04
42	72	2,30E-04	1,27E-04	3,88E-04	2,61E-04	8,27E-05	6,11E-04	6,19E-04

43	73	2,44E-04	4,18E-04	4,30E-04	1,97E-04	6,01E-04	6,37E-04	5,79E-04
44	74	1,60E-04	1,04E-04	2,04E-04	1,50E-04	1,53E-04	1,82E-04	2,20E-04
45	75	2,51E-04	2,55E-04	4,99E-04	2,58E-04	3,53E-04	5,97E-04	
46	76	4,63E-04	6,77E-04	6,66E-04	5,30E-04	1,60E-03	7,71E-04	6,06E-04
47	77	9,05E-05	2,04E-04	3,65E-04	7,10E-05	2,47E-04	6,64E-04	2,04E-04
48	78	3,65E-04	4,54E-04	4,91E-04	5,94E-04	1,06E-03	6,40E-04	8,20E-04
49	79	6,58E-05	1,32E-04	3,31E-04	6,46E-05	1,07E-04	3,27E-04	1,50E-04
50	81	4,29E-05	3,54E-04	5,15E-04	7,54E-05	6,94E-04	5,71E-04	1,16E-04
51	82	1,02E-04	5,89E-04	7,36E-04	1,02E-04	9,34E-04	1,10E-03	2,45E-04
52	83	2,11E-04	9,72E-04	5,68E-04	1,67E-04	1,02E-03	5,26E-04	3,88E-04
53	84	8,30E-05	2,04E-04	2,48E-04	6,96E-05	5,09E-04	6,20E-04	1,42E-04
54	87	4,37E-05	4,10E-04	5,28E-04	3,38E-05	4,92E-04	7,82E-04	8,91E-05
55	89	1,18E-04	2,93E-04	2,36E-04	5,84E-05	7,56E-04	6,07E-04	2,02E-04
56	90	1,66E-04	7,96E-04	6,15E-04	1,49E-04	9,06E-04	8,00E-04	2,96E-04
57	92	3,42E-04	3,13E-04	4,53E-04	3,88E-04	3,96E-04	6,01E-04	5,75E-04
58	93	5,11E-04	6,16E-04	5,91E-04	6,40E-04	8,16E-04	6,76E-04	2,55E-04
59	94	7,96E-05	2,01E-04	3,05E-04	7,01E-05	2,60E-04	4,48E-04	2,01E-04
60	95	5,16E-04	4,95E-04	6,23E-04	5,32E-04	7,29E-04	7,62E-04	7,37E-04
61	96	7,34E-05	2,97E-04	3,26E-04	6,27E-05	5,35E-04	3,66E-04	1,62E-04
62	97	3,37E-04	6,50E-04	4,86E-04	2,75E-04	9,07E-04	6,67E-04	2,13E-04
63	98	1,41E-04	2,20E-04	5,36E-04	1,23E-04	2,60E-04	6,69E-04	5,59E-04
64	99	2,79E-04	4,13E-04	4,33E-04	2,79E-04	6,82E-04	3,77E-04	1,67E-04
65	101	5,10E-05	3,58E-04	7,84E-04	6,93E-05	3,54E-04	6,33E-04	7,40E-05
66	102	1,51E-04	5,94E-04	5,90E-04	1,77E-04	7,71E-04	6,98E-04	3,38E-04
67	103	1,84E-04	6,60E-04	5,03E-04	1,24E-04	7,06E-04	2,88E-04	3,06E-04
68	104	6,78E-05	2,81E-04	8,27E-04	8,15E-05	3,13E-04	6,77E-04	2,28E-04

Code	
M0	Mother at birth
B0	Baby at Birth
B2.5	Baby at 2.5 months
B6	Baby at 6 months
B9	Baby at 9 months
M9	Mother at 9 months

		B6			B9			M9
**MSP2**	**CSP**	**AMA1**	**MSP2**	**CSP**	**AMA1**	**MSP2**	**CSP**	**AMA1**
8,71E-04	7,08E-04	5,11E-04	5,98E-04	4,43E-04	3,05E-04	5,05E-04	5,75E-04	3,56E-04
3,53E-04	4,91E-04	7,90E-04	6,72E-04	4,50E-04	5,91E-04	3,88E-04	4,32E-04	6,31E-05
4,36E-04	5,11E-04	5,78E-04	7,92E-04	4,83E-04	5,96E-04	5,15E-04	3,80E-04	3,39E-04
8,14E-04	4,90E-04	4,17E-04	6,40E-04	4,53E-04	5,64E-04	5,27E-04	3,66E-04	1,62E-04
8,43E-04	4,86E-04	3,01E-04	3,07E-04	3,41E-04	4,15E-04	4,66E-04	4,63E-04	6,56E-04
6,03E-04	2,95E-04	7,35E-04	7,73E-04	4,73E-04	2,58E-04	1,91E-04	2,68E-04	9,65E-05
4,88E-04	4,05E-04	5,43E-04	4,62E-04	3,60E-04	6,99E-04	3,55E-04	4,30E-04	9,92E-05
8,49E-04	4,67E-04	4,37E-04	3,76E-04	3,30E-04	6,30E-04	5,59E-04	4,64E-04	2,68E-05
7,89E-04	5,05E-04	1,00E-03	7,55E-04	5,32E-04	7,29E-04	6,13E-04	5,12E-04	2,11E-04
4,89E-04	4,13E-04	6,95E-04	6,82E-04	3,90E-04	3,65E-04	4,40E-04	4,08E-04	1,74E-04
4,78E-04	4,14E-04	3,59E-04	7,24E-04	5,07E-04	2,76E-04	2,83E-04	2,56E-04	5,47E-05
3,36E-04	4,20E-04	3,36E-04	3,33E-04	3,22E-04	2,90E-04	4,76E-04	4,03E-04	5,99E-05
3,61E-04	3,18E-04	4,49E-04	4,76E-04	4,40E-04	1,10E-04	4,44E-04	4,55E-04	2,90E-05
7,95E-04	6,50E-04	6,72E-04	1,11E-03	9,95E-04	1,00E-03	6,05E-04	6,34E-04	1,28E-04
4,69E-04	3,62E-04	6,68E-04	6,15E-04	4,54E-04	8,91E-04	6,46E-04	5,63E-04	3,78E-04
3,33E-03	1,41E-03	1,62E-03	3,49E-03	1,47E-03	4,14E-04	7,24E-04	3,49E-04	2,23E-04
4,48E-04	2,77E-04	2,42E-04	3,52E-04	1,04E-03	6,47E-04	3,61E-03	1,18E-03	2,31E-04
3,08E-03	6,61E-04	4,73E-04	1,97E-03	1,41E-03	4,70E-04	9,06E-04	6,54E-04	2,29E-04
3,18E-03	7,26E-04	1,59E-03	3,56E-03	1,16E-03	5,87E-04	1,89E-03	4,54E-04	4,07E-04
1,36E-03	5,47E-04	6,16E-04	7,01E-04	4,67E-04	6,93E-04	7,89E-04	5,37E-04	3,56E-04
1,83E-03	8,30E-04	8,59E-04	1,65E-03	7,16E-04	2,03E-04	1,03E-03	6,23E-04	3,95E-05
8,72E-04	5,41E-04	8,92E-04	1,25E-03	6,30E-04	3,83E-04	4,73E-04	3,17E-04	1,04E-04
1,19E-03	5,31E-04	6,20E-04	1,10E-03	5,31E-04	7,60E-04	4,68E-04	5,66E-04	9,37E-05
6,72E-04	4,71E-04	5,63E-04	1,37E-03	6,31E-04	6,63E-04	9,20E-04	5,27E-04	9,34E-05
8,79E-04	5,23E-04	6,18E-04	1,28E-03	5,28E-04	9,47E-04	1,81E-03	9,07E-04	3,10E-04
9,22E-04	5,39E-04	6,45E-04	1,15E-03	6,37E-04	5,17E-04	8,63E-04	6,11E-04	1,21E-04
4,49E-04	6,97E-04	4,44E-04	5,14E-04	4,98E-04	7,01E-04	1,15E-03	6,72E-04	6,91E-04
7,65E-04	6,21E-04	4,16E-04	5,66E-04	5,16E-04	4,79E-04	7,62E-04	5,31E-04	7,55E-05
9,15E-04	5,78E-04	7,80E-04	1,44E-03	6,65E-04	6,73E-04	8,53E-04	5,49E-04	4,48E-04
4,67E-04	4,73E-04	5,58E-04	1,24E-03	5,41E-04	4,74E-05	5,52E-04	3,84E-04	7,34E-05
8,18E-04	5,18E-04	6,29E-04	9,14E-04	5,94E-04	7,16E-05	3,87E-04	5,85E-04	7,50E-05
1,15E-03	5,99E-04	7,85E-05	3,42E-04	2,73E-04	5,66E-04	1,07E-03	8,06E-04	1,17E-04
9,27E-04	5,71E-04	6,14E-04	8,38E-04	5,11E-04	4,01E-05	2,34E-04	4,87E-04	5,21E-05
6,70E-04	6,58E-04				4,81E-04	4,53E-04	3,61E-04	1,29E-04
9,61E-04	6,45E-04	3,07E-04	3,58E-04	3,52E-04	7,71E-05	4,47E-04	6,61E-04	7,99E-05
6,26E-04	5,73E-04	3,17E-04	3,71E-04	3,63E-04	1,58E-04	1,96E-04	6,49E-04	1,54E-04
3,56E-04	6,03E-04	9,58E-05	6,98E-04	4,89E-04	5,59E-05	4,06E-04	5,89E-04	5,92E-05
1,07E-03	5,52E-04	1,38E-04	1,06E-03	4,58E-04	3,73E-04	4,52E-04	3,85E-04	1,20E-04
1,10E-03	6,49E-04	2,75E-04	6,78E-04	6,01E-04	4,14E-04	3,02E-04	2,99E-04	3,51E-05
6,65E-04	5,26E-04	5,63E-04	7,18E-04	5,52E-04	3,95E-04	4,52E-04	3,82E-04	1,90E-04
7,10E-04	5,00E-04	1,68E-04	4,22E-04	6,83E-04	3,06E-04	9,12E-04	6,75E-04	5,18E-05
2,64E-04	5,51E-04	6,36E-04	4,90E-04	5,72E-04	2,98E-04	4,21E-04	2,46E-04	1,36E-04

1,04E-03	6,33E-04	6,62E-04	7,97E-04	6,46E-04	7,32E-05	6,89E-04	6,83E-04	6,80E-05
4,00E-04	4,77E-04	6,82E-04	1,22E-03	7,51E-04	5,51E-04	6,37E-04	5,87E-04	1,15E-04
		6,12E-04	7,42E-04	5,55E-04	3,19E-04	4,10E-04	4,39E-04	2,45E-04
1,05E-03	5,97E-04	3,76E-04	5,29E-04	4,77E-04	6,10E-04	8,45E-04	6,29E-04	2,01E-04
5,53E-04	6,83E-04	6,21E-04	8,63E-04	6,41E-04	5,81E-04	7,03E-04	5,40E-04	1,01E-04
1,40E-03	8,10E-04	1,00E-03	1,39E-03	8,02E-04	4,25E-04	5,47E-04	4,56E-04	1,36E-04
4,50E-04	5,94E-04	5,83E-04	8,29E-04	5,09E-04	6,41E-04	1,18E-03	7,81E-04	6,66E-05
9,39E-04	7,26E-04	4,61E-04	5,92E-04	4,36E-04	7,30E-04	5,16E-04	8,01E-04	1,23E-04
1,01E-03	7,46E-04	1,77E-04	4,33E-04	7,43E-04	8,82E-05	2,52E-04	6,58E-04	1,72E-04
1,30E-03	6,99E-04	6,67E-04	1,23E-03	7,13E-04	4,56E-04	6,08E-04	5,54E-04	2,87E-05
8,22E-04	6,43E-04	8,47E-05	5,81E-04	5,84E-04	4,24E-04	6,67E-04	7,31E-04	3,32E-04
7,69E-04	6,64E-04	6,81E-04	8,73E-04	7,00E-04	3,46E-04	5,62E-04	4,19E-04	1,03E-04
8,47E-04	6,15E-04	3,82E-04	8,45E-04	6,66E-04	6,34E-04	1,46E-03	9,42E-04	4,91E-04
9,67E-04	7,10E-04	4,87E-04	9,92E-04	7,64E-04	5,75E-04	5,77E-04	8,51E-04	1,32E-04
7,21E-04	6,06E-04	1,30E-04	4,23E-04	4,32E-04	4,38E-04	5,18E-04	4,55E-04	2,74E-04
5,19E-04	4,69E-04	8,00E-04	1,04E-03	8,38E-04	2,66E-04	4,94E-04	5,24E-04	7,87E-05
6,65E-04	6,34E-04	3,76E-04	6,35E-04	5,64E-04	3,28E-04	3,65E-04	3,42E-04	9,04E-05
1,09E-03	7,22E-04	1,90E-04	5,76E-04	6,14E-04	7,18E-05	2,87E-04	5,20E-04	8,06E-05
6,26E-04	4,34E-04	4,13E-04	5,57E-04	4,78E-04	8,30E-05	5,54E-04	7,36E-04	2,16E-04
4,08E-04	5,67E-04	8,10E-04	1,06E-03	8,41E-04	4,87E-04	5,80E-04	4,89E-04	3,79E-04
8,97E-04	5,73E-04	8,07E-05	6,81E-04	7,56E-04	3,53E-04	4,32E-04	3,64E-04	3,29E-04
7,58E-04	5,16E-04	3,84E-04	3,73E-04	6,12E-04	1,72E-04	1,92E-04	2,78E-04	1,04E-04
5,36E-04	4,83E-04	6,46E-04	8,50E-04	6,93E-04	1,16E-04	2,02E-04	6,53E-04	3,54E-04
7,46E-04	5,74E-04	5,59E-04	8,57E-04	6,28E-04	9,48E-05	6,46E-04	6,13E-04	3,82E-04
9,06E-04	6,67E-04	5,72E-04	7,70E-04	6,01E-04	9,28E-05	2,17E-04	6,18E-04	2,97E-04
5,85E-04	6,57E-04	2,75E-04	6,41E-04	5,51E-04	2,57E-04	2,42E-04	3,69E-04	3,84E-05

MSP2	CSP
5,02E-04	5,61E-04
5,37E-04	3,12E-04
4,89E-04	4,84E-04
3,82E-04	3,61E-04
5,70E-04	4,38E-04
2,08E-04	3,36E-04
2,24E-04	3,45E-04
2,07E-04	2,69E-04
4,08E-04	3,10E-04
3,82E-04	3,47E-04
2,39E-04	2,76E-04
4,17E-04	2,13E-04
2,08E-04	2,19E-04
3,91E-04	5,56E-04
1,81E-04	7,38E-04
5,97E-04	3,54E-04
4,46E-04	1,01E-03
1,29E-03	6,91E-04
1,69E-03	-2,48E-04
1,79E-04	4,89E-04
2,04E-04	4,25E-04
5,13E-04	3,53E-04
4,17E-04	4,39E-04
6,47E-04	1,72E-04
3,21E-04	1,95E-04
5,56E-04	3,43E-04
1,26E-03	6,72E-04
5,29E-04	1,84E-04
3,69E-04	4,22E-04
4,25E-04	4,18E-04
3,62E-04	5,19E-04
2,95E-04	5,56E-04
2,35E-04	1,19E-04
2,66E-04	3,48E-04
3,57E-04	5,37E-04
1,58E-04	4,64E-04
2,13E-04	8,80E-04
4,37E-04	4,09E-04
4,15E-04	1,33E-04
1,65E-04	2,25E-04
2,08E-04	2,15E-04
4,13E-04	5,50E-04

3,96E-04	4,09E-04
3,06E-04	4,42E-04
4,49E-04	5,65E-04
4,34E-04	4,01E-04
1,74E-04	4,53E-04
7,69E-04	7,12E-04
1,31E-04	3,11E-04
1,61E-04	6,57E-04
2,54E-04	2,60E-04
3,67E-04	4,50E-04
5,16E-04	4,37E-04
2,50E-04	5,25E-04
7,38E-04	7,02E-04
2,79E-04	3,46E-04
4,84E-04	4,81E-04
1,79E-04	2,42E-04
1,52E-04	3,75E-04
3,16E-04	3,94E-04
2,03E-04	3,96E-04
5,53E-04	4,88E-04
4,63E-04	3,46E-04
5,43E-04	4,65E-04
5,66E-04	5,25E-04
6,93E-04	4,37E-04
4,09E-04	3,72E-04
2,46E-04	4,81E-04
