# Supplementary material for: Development of high affinity antibodies to Plasmodium falciparum merozoite and sporozoite antigens during infancy and adulthood
Source: Front Immunol. 2025 Jul 2;16:1562671. doi: 10.3389/fimmu.2025.1562671 (PMC12263619; doi:10.3389/fimmu.2025.1562671)
Supplement: Supplementary file 2 [file DataSheet2.pdf]

Pearson correlations Infants at birth

Variable		rho	Adj,p-val*
Atypical MBC	Naive B cells	-0.332	0.004
	Plasma cells/blasts	0.014	0.965
	Total frequency of B cells	-0.224	0.097
	Pf+ IgG MBC	0.577	<0.001
	Pf+ non-IgG MBC	0.064	0.808
	Pf+ Atypical MBC	0.928	<0.001
	Pf+ Naive B cells	-0.321	0.006
	Pf+ Plasma cells/blasts	0.012	0.971
	Total frequency of Pf+ B cells	0.042	0.871
	Schizont-spec IgG	0.246	0.081
	Schizont-spec IgM	0.790	<0.001
	AMA1	-0.119	0.706
	MSP2	-0.141	0.630
	CSP	0.071	0.856
IgG MBC	non-IgG MBC	0.318	0.007
	Atypical MBC	0.624	<0.001
	Naive B cells	-0.588	<0.001
	Plasma cells/blasts	0.278	0.025
	Total frequency of B cells	-0.244	0.061
	Pf+ IgG MBC	0.851	<0.001
	Pf+ non-IgG MBC	0.288	0.019
	Pf+ Atypical MBC	0.566	<0.001
	Pf+ Naive B cells	-0.627	<0.001
	Pf+ Plasma cells/blasts	0.249	0.056
	Total frequency of Pf+ B cells	0.364	0.001
	Schizont-spec IgG	-0.001	0.998
	Schizont-spec IgM	0.295	0.078
	AMA1	-0.111	0.742
	MSP2	-0.097	0.786
	CSP	-0.015	0.974
Naive B cells	Plasma cells/blasts	-0.883	<0.001
	Total frequency of B cells	0.223	0.097
	Pf+ IgG MBC	-0.435	<0.001
	Pf+ non-IgG MBC	-0.222	0.100
	Pf+ Atypical MBC	-0.283	0.022
	Pf+ Naive B cells	0.879	<0.001
	Pf+ Plasma cells/blasts	-0.863	<0.001
	Total frequency of Pf+ B cells	-0.626	<0.001
	Schizont-spec IgG	0.045	0.871

	Schizont-spec IgM	-0.230	0.221
	AMA1	0.122	0.700
	MSP2	-0.046	0.894
	CSP	0.035	0.925
non-IgG MBC	Atypical MBC	0.043	0.871
	Naive B cells	-0.355	0.002
	Plasma cells/blasts	0.042	0.871
	Total frequency of B cells	0.047	0.862
	Pf+ IgG MBC	0.342	0.003
	Pf+ non-IgG MBC	0.816	<0.001
	Pf+ Atypical MBC	-0.004	0.986
	Pf+ Naive B cells	-0.576	<0.001
	Pf+ Plasma cells/blasts	0.038	0.875
	Total frequency of Pf+ B cells	0.622	<0.001
	Schizont-spec IgG	0.022	0.945
	Schizont-spec IgM	0.001	0.998
	AMA1	-0.121	0.700
	MSP2	0.139	0.640
	CSP	-0.126	0.686
Pf+ Atypical MBC	Pf+ Naive B cells	-0.267	0.033
	Pf+ Plasma cells/blasts	-0.007	0.983
	Total frequency of Pf+ B cells	0.022	0.940
	Schizont-spec IgG	0.182	0.260
	Schizont-spec IgM	0.682	<0.001
	AMA1	-0.075	0.845
	MSP2	-0.138	0.640
	CSP	0.081	0.832
Pf+ IgG MBC	Pf+ non-IgG MBC	0.388	<0.001
	Pf+ Atypical MBC	0.455	<0.001
	Pf+ Naive B cells	-0.564	<0.001
	Pf+ Plasma cells/blasts	0.140	0.424
	Total frequency of Pf+ B cells	0.234	0.073
	Schizont-spec IgG	0.051	0.858
	Schizont-spec IgM	0.388	0.010
	AMA1	-0.100	0.772
	MSP2	-0.059	0.871
	CSP	-0.077	0.842
Pf+ Naive B cells	Pf+ Plasma cells/blasts	-0.664	<0.001
	Total frequency of Pf+ B cells	-0.512	<0.001
	Schizont-spec IgG	-0.010	0.975

	Schizont-spec IgM	-0.194	0.357
	AMA1	0.103	0.762
	MSP2	-0.202	0.392
	CSP	0.075	0.845
	Pf+ Atypical MBC	-0.019	0.951
	Pf+ Naive B cells	-0.628	<0.001
	Pf+ Plasma cells/blasts	-0.041	0.872
	Total frequency of Pf+ B cells	0.241	0.063
	Schizont-spec IgG	0.111	0.611
	Schizont-spec IgM	0.010	0.983
	AMA1	-0.054	0.875
	MSP2	0.304	0.103
	CSP	-0.117	0.713
Pf+ Plasma cells/blasts	Total frequency of Pf+ B cells	0.409	<0.001
	Schizont-spec IgG	-0.114	0.594
	Schizont-spec IgM	0.010	0.983
	AMA1	-0.082	0.828
	MSP2	0.072	0.855
	CSP	0.008	0.983
Plasma cells/blasts	Total frequency of B cells	-0.186	0.201
	Pf+ IgG MBC	0.145	0.392
	Pf+ non-IgG MBC	-0.046	0.864
	Pf+ Atypical MBC	-0.006	0.983
	Pf+ Naive B cells	-0.676	<0.001
	Pf+ Plasma cells/blasts	0.995	<0.001
	Total frequency of Pf+ B cells	0.444	<0.001
	Schizont-spec IgG	-0.119	0.567
	Schizont-spec IgM	0.012	0.975
	AMA1	-0.075	0.846
	MSP2	0.064	0.864
	CSP	0.001	0.998
Schizont-spec IgG	Schizont-spec IgM	0.744	<0.001
	AMA1	-0.071	0.853
	MSP2	-0.084	0.813
	CSP	-0.006	0.986
Schizont-spec IgG	AMA1	-0.052	0.894
	MSP2	-0.140	0.694
	CSP	0.075	0.858
Total frequency of B cells	Pf+ IgG MBC	-0.211	0.121
	Pf+ non-IgG MBC	0.043	0.871
	Pf+ Atypical MBC	-0.179	0.226

	Pf+ Naive B cells	0.175	0.239
	Pf+ Plasma cells/blasts	-0.183	0.209
	Total frequency of Pf+ B cells	-0.138	0.434
	Schizont-spec IgG	-0.066	0.812
	Schizont-spec IgM	-0.052	0.872
	AMA1	0.053	0.877
	MSP2	-0.030	0.941
	CSP	0.048	0.894
Total frequency of Pf+ B cells	Schizont-spec IgG	-0.072	0.790
	Schizont-spec IgM	0.086	0.790
	AMA1	-0.171	0.508
	MSP2	-0.076	0.844
	CSP	-0.123	0.700

*Adjusted with false discovery rate (FDR).

Pearson correlations babies at 2.5 months.

**Variable**		**rho**	**Adj,p-val***
Atypical MBC	Naive B cells	-0.123	0.557
	Plasma cells/blasts	-0.097	0.691
	Total frequency of B cells	-0.108	0.640
	Pf+ IgG MBC	0.464	<0.001
	Pf+ non-IgG MBC	-0.146	0.453
	Pf+ Atypical MBC	0.627	<0.001
	Pf+ Naive B cells	-0.045	0.871
	Pf+ Plasma cells/blasts	-0.121	0.566
	Total frequency of Pf+ B cells	0.322	0.014
	Schizont-spec IgG	-0.056	0.856
	Schizont-spec IgM	-0.034	0.925
	AMA1	0.041	0.907
	MSP2	-0.006	0.986
	CSP	0.153	0.577
IgG MBC	non-IgG MBC	0.262	0.063
	Atypical MBC	0.529	<0.001
	Naive B cells	-0.355	0.005
	Plasma cells/blasts	0.036	0.894
	Total frequency of B cells	-0.233	0.113
	Pf+ IgG MBC	0.939	<0.001
	Pf+ non-IgG MBC	0.081	0.757
	Pf+ Atypical MBC	-0.021	0.950

	Pf+ Naive B cells	-0.293	0.030
	Pf+ Plasma cells/blasts	0.027	0.925
	Total frequency of Pf+ B cells	0.593	<0.001
	Schizont-spec IgG	-0.058	0.853
	Schizont-spec IgM	-0.110	0.725
	AMA1	-0.102	0.763
	MSP2	-0.139	0.638
	CSP	-0.115	0.723
Naive B cells	Plasma cells/blasts	-0.780	<0.001
	Total frequency of B cells	0.508	<0.001
	Pf+ IgG MBC	-0.288	0.034
	Pf+ non-IgG MBC	-0.265	0.059
	Pf+ Atypical MBC	-0.034	0.901
	Pf+ Naive B cells	0.847	<0.001
	Pf+ Plasma cells/blasts	-0.762	<0.001
	Total frequency of Pf+ B cells	-0.593	<0.001
	Schizont-spec IgG	0.063	0.836
	Schizont-spec IgM	0.053	0.875
	AMA1	-0.061	0.868
	MSP2	-0.054	0.875
	CSP	0.163	0.540
non-IgG MBC	Atypical MBC	-0.023	0.944
	Naive B cells	-0.421	<0.001
	Plasma cells/blasts	-0.083	0.753
	Total frequency of B cells	-0.385	0.002
	Pf+ IgG MBC	0.197	0.219
	Pf+ non-IgG MBC	0.801	<0.001
	Pf+ Atypical MBC	-0.079	0.763
	Pf+ Naive B cells	-0.557	<0.001
	Pf+ Plasma cells/blasts	-0.119	0.579
	Total frequency of Pf+ B cells	0.513	<0.001
	Schizont-spec IgG	-0.042	0.875
	Schizont-spec IgM	0.101	0.756
	AMA1	0.120	0.706
	MSP2	0.089	0.808
	CSP	-0.248	0.229
Pf+ Atypical MBC	Pf+ Naive B cells	-0.102	0.670
	Pf+ Plasma cells/blasts	-0.141	0.476
	Total frequency of Pf+ B cells	-0.028	0.925
	Schizont-spec IgG	-0.060	0.834

	Schizont-spec IgM	0.042	0.894
	AMA1	0.117	0.713
	MSP2	0.162	0.544
	CSP	0.242	0.246
Pf+ IgG MBC	Pf+ non-IgG MBC	0.102	0.667
	Pf+ Atypical MBC	-0.032	0.910
	Pf+ Naive B cells	-0.254	0.071
	Pf+ Plasma cells/blasts	0.013	0.972
	Total frequency of Pf+ B cells	0.486	<0.001
	Schizont-spec IgG	-0.051	0.863
	Schizont-spec IgM	-0.096	0.769
	AMA1	-0.116	0.712
	MSP2	-0.178	0.476
	CSP	-0.127	0.682
Pf+ Naive B cells	Pf+ Plasma cells/blasts	-0.479	<0.001
	Total frequency of Pf+ B cells	-0.378	0.002
	Schizont-spec IgG	0.093	0.709
	Schizont-spec IgM	-0.041	0.901
	AMA1	-0.051	0.879
	MSP2	-0.061	0.868
	CSP	0.210	0.357
Pf+ non-IgG MBC	Pf+ Atypical MBC	0.049	0.858
	Pf+ Naive B cells	-0.574	<0.001
	Pf+ Plasma cells/blasts	-0.189	0.241
	Total frequency of Pf+ B cells	0.125	0.548
	Schizont-spec IgG	-0.053	0.856
	Schizont-spec IgM	-0.015	0.971
	AMA1	0.124	0.688
	MSP2	0.137	0.640
	CSP	-0.129	0.673
Pf+ Plasma cells/blasts	Total frequency of Pf+ B cells	0.266	0.059
	Schizont-spec IgG	-0.007	0.983
	Schizont-spec IgM	0.019	0.965
	AMA1	-0.015	0.974
	MSP2	-0.035	0.925
	CSP	-0.124	0.686
Plasma cells/blasts	Total frequency of B cells	-0.263	0.061
	Pf+ IgG MBC	0.004	0.986
	Pf+ non-IgG MBC	-0.156	0.401
	Pf+ Atypical MBC	-0.123	0.557

	Pf+ Naive B cells	-0.528	<0.001
	Pf+ Plasma cells/blasts	0.974	<0.001
	Total frequency of Pf+ B cells	0.315	0.016
	Schizont-spec IgG	0.005	0.986
	Schizont-spec IgM	-0.111	0.720
	AMA1	-0.022	0.958
	MSP2	-0.020	0.965
	CSP	-0.111	0.742
Schizont-spec IgG	Schizont-spec IgM	-0.085	0.790
	AMA1	0.024	0.956
	MSP2	0.046	0.894
	CSP	0.126	0.686
Schizont-spec IgM	AMA1	-0.097	0.812
	MSP2	-0.118	0.756
	CSP	-0.171	0.581
Total frequency of B cells	Pf+ IgG MBC	-0.185	0.255
	Pf+ non-IgG MBC	-0.356	0.004
	Pf+ Atypical MBC	-0.084	0.751
	Pf+ Naive B cells	0.607	<0.001
	Pf+ Plasma cells/blasts	-0.259	0.064
	Total frequency of Pf+ B cells	-0.173	0.316
	Schizont-spec IgG	0.180	0.284
	Schizont-spec IgM	0.022	0.958
	AMA1	0.055	0.875
	MSP2	-0.036	0.925
	CSP	0.298	0.108
Total frequency of Pf+ B cells	Schizont-spec IgG	0.013	0.971
	Schizont-spec IgM	0.014	0.974
	AMA1	-0.011	0.982
	MSP2	0.023	0.958
	CSP	-0.175	0.496

*Adjusted with false discovery rate (FDR).

Pearson correlations babies at 6 months.

Variable		rho	Adj,p-val*
Atypical MBC	Naive B cells	-0.211	0.140
	Plasma cells/blasts	-0.051	0.856
	Total frequency of B cells	-0.155	0.371
	Pf+ IgG MBC	0.413	<0.001

	Pf+ non-IgG MBC	-0.133	0.476
	Pf+ Atypical MBC	0.887	<0.001
	Pf+ Naive B cells	-0.137	0.459
	Pf+ Plasma cells/blasts	-0.052	0.856
	Total frequency of Pf+ B cells	0.290	0.022
	Schizont-spec IgG	0.067	0.808
	Schizont-spec IgM	0.160	0.496
	AMA1	-0.071	0.856
	MSP2	-0.186	0.466
	CSP	-0.087	0.812
IgG MBC	non-IgG MBC	0.357	0.002
	Atypical MBC	0.482	<0.001
	Naive B cells	-0.390	0.001
	Plasma cells/blasts	0.130	0.493
	Total frequency of B cells	-0.189	0.212
	Pf+ IgG MBC	0.912	<0.001
	Pf+ non-IgG MBC	0.123	0.521
	Pf+ Atypical MBC	0.245	0.067
	Pf+ Naive B cells	-0.294	0.019
	Pf+ Plasma cells/blasts	0.115	0.561
	Total frequency of Pf+ B cells	0.385	0.001
	Schizont-spec IgG	-0.023	0.941
	Schizont-spec IgM	0.126	0.642
	AMA1	-0.020	0.965
	MSP2	-0.084	0.823
	CSP	-0.074	0.853
Naive B cells	Plasma cells/blasts	-0.722	<0.001
	Total frequency of B cells	0.302	0.016
	Pf+ IgG MBC	-0.245	0.067
	Pf+ non-IgG MBC	-0.089	0.704
	Pf+ Atypical MBC	-0.043	0.871
	Pf+ Naive B cells	0.791	<0.001
	Pf+ Plasma cells/blasts	-0.687	<0.001
	Total frequency of Pf+ B cells	-0.572	<0.001
	Schizont-spec IgG	0.079	0.756
	Schizont-spec IgM	-0.108	0.712
	AMA1	-0.127	0.686
	MSP2	-0.069	0.856
	CSP	0.080	0.838
non-IgG MBC	Atypical MBC	-0.007	0.983
	Naive B cells	-0.343	0.004

	Plasma cells/blasts	0.039	0.877
	Total frequency of B cells	-0.159	0.348
	Pf+ IgG MBC	0.248	0.064
	Pf+ non-IgG MBC	0.817	<0.001
	Pf+ Atypical MBC	-0.196	0.182
	Pf+ Naive B cells	-0.379	0.001
	Pf+ Plasma cells/blasts	0.007	0.983
	Total frequency of Pf+ B cells	0.610	<0.001
	Schizont-spec IgG	-0.018	0.957
	Schizont-spec IgM	0.054	0.871
	AMA1	0.277	0.159
	MSP2	0.176	0.499
	CSP	-0.021	0.965
Pf+ Atypical MBC	Pf+ Naive B cells	-0.016	0.958
	Pf+ Plasma cells/blasts	-0.126	0.505
	Total frequency of Pf+ B cells	0.213	0.135
	Schizont-spec IgG	0.045	0.871
	Schizont-spec IgM	0.175	0.438
	AMA1	-0.183	0.476
	MSP2	-0.248	0.237
	CSP	-0.160	0.557
Pf+ IgG MBC	Pf+ non-IgG MBC	0.111	0.582
	Pf+ Atypical MBC	0.252	0.059
	Pf+ Naive B cells	-0.220	0.113
	Pf+ Plasma cells/blasts	0.040	0.875
	Total frequency of Pf+ B cells	0.281	0.027
	Schizont-spec IgG	-0.038	0.883
	Schizont-spec IgM	0.139	0.581
	AMA1	-0.048	0.894
	MSP2	-0.107	0.754
	CSP	-0.107	0.756
Pf+ Naive B cells	Pf+ Plasma cells/blasts	-0.527	<0.001
	Total frequency of Pf+ B cells	-0.399	<0.001
	Schizont-spec IgG	0.096	0.686
	Schizont-spec IgM	-0.060	0.863
	AMA1	-0.120	0.709
	MSP2	0.016	0.972
	CSP	0.072	0.856
Pf+ non-IgG MBC	Pf+ Atypical MBC	-0.251	0.059
	Pf+ Naive B cells	-0.333	0.006

	Pf+ Plasma cells/blasts	-0.105	0.612
	Total frequency of Pf+ B cells	0.287	0.023
	Schizont-spec IgG	0.018	0.955
	Schizont-spec IgM	0.014	0.973
	AMA1	0.228	0.283
	MSP2	0.107	0.753
	CSP	-0.041	0.906
Pf+ Plasma cells/blasts	Total frequency of Pf+ B cells	0.406	<0.001
	Schizont-spec IgG	0.001	0.998
	Schizont-spec IgM	0.042	0.894
	AMA1	0.059	0.871
	MSP2	0.061	0.868
	CSP	-0.106	0.753
Plasma cells/blasts	Total frequency of B cells	-0.120	0.544
	Pf+ IgG MBC	0.040	0.875
	Pf+ non-IgG MBC	-0.121	0.536
	Pf+ Atypical MBC	-0.123	0.522
	Pf+ Naive B cells	-0.546	<0.001
	Pf+ Plasma cells/blasts	0.985	<0.001
	Total frequency of Pf+ B cells	0.456	<0.001
	Schizont-spec IgG	-0.052	0.856
	Schizont-spec IgM	0.079	0.812
	AMA1	0.038	0.919
	MSP2	0.063	0.868
	CSP	-0.079	0.842
Schizont-spec IgG	Schizont-spec IgM	0.071	0.839
	AMA1	0.034	0.925
	MSP2	-0.066	0.858
	CSP	-0.144	0.608
Schizont-spec IgM	AMA1	0.034	0.940
	MSP2	0.004	0.992
	CSP	-0.054	0.894
Total frequency of B cells	Pf+ IgG MBC	-0.205	0.159
	Pf+ non-IgG MBC	-0.127	0.505
	Pf+ Atypical MBC	-0.127	0.505
	Pf+ Naive B cells	0.401	<0.001
	Pf+ Plasma cells/blasts	-0.138	0.459
	Total frequency of Pf+ B cells	-0.238	0.081
	Schizont-spec IgG	-0.172	0.300
	Schizont-spec IgM	0.086	0.790

	AMA1	-0.082	0.832
	MSP2	-0.096	0.790
	CSP	0.093	0.800
Total frequency of Pf+ B cells	Schizont-spec IgG	-0.036	0.894
	Schizont-spec IgM	0.253	0.156
	AMA1	0.344	0.058
	MSP2	0.288	0.137
	CSP	0.027	0.950

*Adjusted with false discovery rate (FDR).

Pearson correlations babies at 9 months.

Variable		rho	Adj,p-val*
Atypical MBC	Naive B cells	-0.481	<0.001
	Plasma cells/blasts	-0.057	0.842
	Total frequency of B cells	-0.497	<0.001
	Pf+ IgG MBC	0.667	<0.001
	Pf+ non-IgG MBC	-0.073	0.774
	Pf+ Atypical MBC	0.922	<0.001
	Pf+ Naive B cells	-0.357	0.002
	Pf+ Plasma cells/blasts	-0.111	0.582
	Total frequency of Pf+ B cells	0.023	0.939
	Schizont-spec IgG	-0.058	0.844
	Schizont-spec IgM	0.032	0.925
	AMA1	-0.193	0.438
	MSP2	-0.360	0.042
	CSP	-0.314	0.094
IgG MBC	non-IgG MBC	0.390	0.001
	Atypical MBC	0.653	<0.001
	Naive B cells	-0.511	<0.001
	Plasma cells/blasts	0.130	0.493
	Total frequency of B cells	-0.311	0.011
	Pf+ IgG MBC	0.878	<0.001
	Pf+ non-IgG MBC	0.119	0.544
	Pf+ Atypical MBC	0.495	<0.001
	Pf+ Naive B cells	-0.385	0.001
	Pf+ Plasma cells/blasts	-0.006	0.983
	Total frequency of Pf+ B cells	0.323	0.008
	Schizont-spec IgG	-0.063	0.828
	Schizont-spec IgM	0.079	0.812

	AMA1	-0.110	0.749
	MSP2	-0.187	0.459
	CSP	-0.096	0.790
Naive B cells	Plasma cells/blasts	-0.762	<0.001
	Total frequency of B cells	0.194	0.190
	Pf+ IgG MBC	-0.484	<0.001
	Pf+ non-IgG MBC	0.020	0.950
	Pf+ Atypical MBC	-0.387	0.001
	Pf+ Naive B cells	0.849	<0.001
	Pf+ Plasma cells/blasts	-0.523	<0.001
	Total frequency of Pf+ B cells	-0.392	0.001
	Schizont-spec IgG	0.077	0.769
	Schizont-spec IgM	-0.281	0.100
	AMA1	-0.223	0.318
	MSP2	-0.089	0.810
	CSP	0.050	0.891
non-IgG MBC	Atypical MBC	0.075	0.763
	Naive B cells	-0.301	0.016
	Plasma cells/blasts	0.135	0.468
	Total frequency of B cells	0.130	0.493
	Pf+ IgG MBC	0.402	<0.001
	Pf+ non-IgG MBC	0.716	<0.001
	Pf+ Atypical MBC	-0.006	0.983
	Pf+ Naive B cells	-0.392	0.001
	Pf+ Plasma cells/blasts	0.089	0.700
	Total frequency of Pf+ B cells	0.699	<0.001
	Schizont-spec IgG	-0.002	0.993
	Schizont-spec IgM	0.117	0.686
	AMA1	0.386	0.024
	MSP2	0.344	0.058
	CSP	0.099	0.780
Pf+ Atypical MBC	Pf+ Naive B cells	-0.286	0.024
	Pf+ Plasma cells/blasts	-0.127	0.505
	Total frequency of Pf+ B cells	0.022	0.940
	Schizont-spec IgG	-0.003	0.989
	Schizont-spec IgM	0.073	0.836
	AMA1	-0.167	0.533
	MSP2	-0.343	0.059
	CSP	-0.281	0.151
Pf+ IgG MBC	Pf+ non-IgG MBC	0.198	0.176
	Pf+ Atypical MBC	0.486	<0.001

	Pf+ Naive B cells	-0.412	<0.001
	Pf+ Plasma cells/blasts	-0.031	0.906
	Total frequency of Pf+ B cells	0.276	0.032
	Schizont-spec IgG	-0.059	0.842
	Schizont-spec IgM	0.102	0.742
	AMA1	-0.039	0.916
	MSP2	-0.182	0.476
	CSP	-0.165	0.540
Pf+ Naive B cells	Pf+ Plasma cells/blasts	-0.620	<0.001
	Total frequency of Pf+ B cells	-0.285	0.025
	Schizont-spec IgG	0.045	0.871
	Schizont-spec IgM	-0.431	0.003
	AMA1	-0.191	0.446
	MSP2	-0.041	0.910
	CSP	-0.009	0.983
Pf+ non-IgG MBC	Pf+ Atypical MBC	-0.182	0.236
	Pf+ Naive B cells	-0.176	0.258
	Pf+ Plasma cells/blasts	-0.197	0.179
	Total frequency of Pf+ B cells	0.216	0.124
	Schizont-spec IgG	0.066	0.812
	Schizont-spec IgM	<0.001	0.998
	AMA1	0.133	0.669
	MSP2	0.185	0.467
	CSP	-0.112	0.743
Pf+ Plasma cells/blasts	Total frequency of Pf+ B cells	0.108	0.600
	Schizont-spec IgG	-0.075	0.778
	Schizont-spec IgM	-0.101	0.743
	AMA1	0.201	0.406
	MSP2	0.077	0.844
	CSP	0.183	0.476
Plasma cells/blasts	Total frequency of B cells	0.031	0.904
	Pf+ IgG MBC	0.078	0.756
	Pf+ non-IgG MBC	-0.132	0.482
	Pf+ Atypical MBC	-0.081	0.745
	Pf+ Naive B cells	-0.631	<0.001
	Pf+ Plasma cells/blasts	0.798	<0.001
	Total frequency of Pf+ B cells	0.318	0.009
	Schizont-spec IgG	-0.045	0.871
	Schizont-spec IgM	-0.028	0.940
	AMA1	0.268	0.182

	MSP2	0.205	0.389
	CSP	0.090	0.808
Schizont-spec IgG	Schizont-spec IgM	-0.125	0.645
	AMA1	-0.054	0.875
	MSP2	-0.072	0.853
	CSP	0.051	0.879
Schizont-spec IgM	AMA1	0.070	0.868
	MSP2	-0.291	0.197
	CSP	-0.524	0.002
Total frequency of B cells	Pf+ IgG MBC	-0.326	0.007
	Pf+ non-IgG MBC	0.157	0.356
	Pf+ Atypical MBC	-0.424	<0.001
	Pf+ Naive B cells	0.227	0.100
	Pf+ Plasma cells/blasts	-0.028	0.916
	Total frequency of Pf+ B cells	0.151	0.387
	Schizont-spec IgG	0.049	0.864
	Schizont-spec IgM	0.042	0.895
	AMA1	0.228	0.301
	MSP2	0.285	0.144
	CSP	-0.016	0.972
Total frequency of Pf+ B cells	Schizont-spec IgG	0.016	0.965
	Schizont-spec IgM	0.236	0.204
	AMA1	0.332	0.068
	MSP2	0.341	0.060
	CSP	0.155	0.576

*Adjusted with false discovery rate (FDR).

Pearson correlations mothers at delivery.

Variable		rho	Adj,p-val*
Atypical MBC	Naive B cells	-0.457	<0.001
	Plasma cells/blasts	-0.265	0.038
	Total frequency of B cells	-0.063	0.790
	Pf+ IgG MBC	0.296	0.016
	Pf+ non-IgG MBC	-0.235	0.080
	Pf+ Atypical MBC	0.765	<0.001
	Pf+ Naive B cells	-0.272	0.032
	Pf+ Plasma cells/blasts	-0.303	0.013
	Total frequency of Pf+ B cells	0.176	0.254
	Schizont-spec IgG	0.051	0.836

	Schizont-spec IgM	0.092	0.761
	AMA1	-0.069	0.831
	MSP2	-0.007	0.977
	CSP	-0.196	0.414
IgG MBC	non-IgG MBC	0.317	0.009
	Atypical MBC	0.280	0.026
	Naive B cells	-0.521	<0.001
	Plasma cells/blasts	0.038	0.877
	Total frequency of B cells	-0.005	0.977
	Pf+ IgG MBC	0.884	<0.001
	Pf+ non-IgG MBC	0.052	0.823
	Pf+ Atypical MBC	0.216	0.119
	Pf+ Naive B cells	-0.442	<0.001
	Pf+ Plasma cells/blasts	0.036	0.877
	Total frequency of Pf+ B cells	0.534	<0.001
	Schizont-spec IgG	0.123	0.544
	Schizont-spec IgM	0.228	0.253
	AMA1	-0.165	0.532
	MSP2	-0.025	0.945
	CSP	0.092	0.787
Naive B cells	Plasma cells/blasts	-0.613	<0.001
	Total frequency of B cells	0.213	0.124
	Pf+ IgG MBC	-0.496	<0.001
	Pf+ non-IgG MBC	0.064	0.789
	Pf+ Atypical MBC	-0.296	0.016
	Pf+ Naive B cells	0.856	<0.001
	Pf+ Plasma cells/blasts	-0.560	<0.001
	Total frequency of Pf+ B cells	-0.524	<0.001
	Schizont-spec IgG	-0.105	0.628
	Schizont-spec IgM	-0.146	0.547
	AMA1	0.163	0.537
	MSP2	-0.045	0.894
	CSP	0.129	0.660
non-IgG MBC	Atypical MBC	-0.039	0.874
	Naive B cells	-0.183	0.230
	Plasma cells/blasts	-0.139	0.420
	Total frequency of B cells	0.056	0.810
	Pf+ IgG MBC	0.461	<0.001
	Pf+ non-IgG MBC	0.788	<0.001
	Pf+ Atypical MBC	-0.246	0.064
	Pf+ Naive B cells	-0.349	0.002

	Pf+ Plasma cells/blasts	-0.175	0.257
	Total frequency of Pf+ B cells	0.316	0.009
	Schizont-spec IgG	0.373	0.002
	Schizont-spec IgM	0.193	0.362
	AMA1	0.015	0.966
	MSP2	0.252	0.239
	CSP	0.091	0.788
Pf+ Atypical MBC	Pf+ Naive B cells	-0.205	0.153
	Pf+ Plasma cells/blasts	-0.293	0.018
	Total frequency of Pf+ B cells	0.136	0.430
	Schizont-spec IgG	-0.099	0.660
	Schizont-spec IgM	0.027	0.931
	AMA1	-0.133	0.648
	MSP2	-0.159	0.547
	CSP	-0.253	0.237
Pf+ IgG MBC	Pf+ non-IgG MBC	0.240	0.073
	Pf+ Atypical MBC	0.164	0.298
	Pf+ Naive B cells	-0.542	<0.001
	Pf+ Plasma cells/blasts	-0.030	0.897
	Total frequency of Pf+ B cells	0.558	<0.001
	Schizont-spec IgG	0.150	0.415
	Schizont-spec IgM	0.219	0.267
	AMA1	-0.041	0.898
	MSP2	0.103	0.746
	CSP	0.201	0.394
Pf+ Naive B cells	Pf+ Plasma cells/blasts	-0.526	<0.001
	Total frequency of Pf+ B cells	-0.472	<0.001
	Schizont-spec IgG	-0.039	0.877
	Schizont-spec IgM	-0.012	0.969
	AMA1	-0.006	0.977
	MSP2	-0.076	0.810
	CSP	-0.045	0.894
Pf+ non-IgG MBC	Pf+ Atypical MBC	-0.469	<0.001
	Pf+ Naive B cells	-0.285	0.022
	Pf+ Plasma cells/blasts	-0.126	0.493
	Total frequency of Pf+ B cells	0.037	0.877
	Schizont-spec IgG	0.247	0.087
	Schizont-spec IgM	0.041	0.894
	AMA1	0.104	0.744
	MSP2	0.329	0.077

	CSP	0.304	0.112
Pf+ Plasma cells/blasts	Total frequency of Pf+ B cells	0.191	0.202
	Schizont-spec IgG	-0.067	0.790
	Schizont-spec IgM	-0.036	0.905
	AMA1	-0.058	0.870
	MSP2	-0.021	0.950
	CSP	-0.034	0.921
Plasma cells/blasts	Total frequency of B cells	-0.215	0.120
	Pf+ IgG MBC	-0.019	0.945
	Pf+ non-IgG MBC	-0.147	0.379
	Pf+ Atypical MBC	-0.262	0.041
	Pf+ Naive B cells	-0.540	<0.001
	Pf+ Plasma cells/blasts	0.974	<0.001
	Total frequency of Pf+ B cells	0.259	0.045
	Schizont-spec IgG	-0.052	0.834
	Schizont-spec IgM	-0.011	0.972
	AMA1	-0.092	0.787
	MSP2	-0.025	0.945
	CSP	-0.056	0.873
Schizont-spec IgG	Schizont-spec IgM	0.234	0.221
	AMA1	-0.144	0.589
	MSP2	0.028	0.939
	CSP	-0.189	0.422
Schizont-spec IgM	AMA1	-0.130	0.706
	MSP2	-0.089	0.810
	CSP	-0.253	0.309
Total frequency of B cells	Pf+ IgG MBC	0.030	0.897
	Pf+ non-IgG MBC	0.156	0.329
	Pf+ Atypical MBC	-0.111	0.560
	Pf+ Naive B cells	0.172	0.266
	Pf+ Plasma cells/blasts	-0.223	0.104
	Total frequency of Pf+ B cells	-0.064	0.790
	Schizont-spec IgG	0.075	0.773
	Schizont-spec IgM	0.103	0.713
	AMA1	-0.190	0.430
	MSP2	-0.179	0.484
	CSP	-0.010	0.975
Total frequency of Pf+ B cells	Schizont-spec IgG	0.252	0.080
	Schizont-spec IgM	0.338	0.037
	AMA1	-0.078	0.810

	MSP2	0.098	0.773
	CSP	0.021	0.950

*Adjusted with false discovery rate (FDR).

Pearson correlations mothers at 9 months postpartum

**Variable**		**rho**	**Adj,p-val***
Atypical MBC	Naive B cells	-0.270	0.042
	Plasma cells/blasts	-0.264	0.049
	Total frequency of B cells	-0.229	0.108
	Pf+ IgG MBC	0.296	0.022
	Pf+ non-IgG MBC	-0.016	0.950
	Pf+ Atypical MBC	0.891	<0.001
	Pf+ Naive B cells	-0.170	0.298
	Pf+ Plasma cells/blasts	-0.342	0.005
	Total frequency of Pf+ B cells	-0.026	0.920
	Schizont-spec IgG	-0.089	0.706
	Schizont-spec IgM	-0.207	0.307
	AMA1	-0.326	0.088
	MSP2	-0.189	0.449
	CSP	0.012	0.972
IgG MBC	non-IgG MBC	0.720	<0.001
	Atypical MBC	0.382	0.001
	Naive B cells	-0.422	<0.001
	Plasma cells/blasts	-0.031	0.897
	Total frequency of B cells	-0.141	0.430
	Pf+ IgG MBC	0.835	<0.001
	Pf+ non-IgG MBC	0.494	<0.001
	Pf+ Atypical MBC	0.234	0.099
	Pf+ Naive B cells	-0.444	<0.001
	Pf+ Plasma cells/blasts	-0.118	0.547
	Total frequency of Pf+ B cells	0.326	0.009
	Schizont-spec IgG	0.058	0.817
	Schizont-spec IgM	0.141	0.568
	AMA1	-0.223	0.328
	MSP2	-0.262	0.226
	CSP	-0.166	0.537
Naive B cells	Plasma cells/blasts	-0.600	<0.001
	Total frequency of B cells	-0.371	0.002
	Pf+ IgG MBC	-0.351	0.004
	Pf+ non-IgG MBC	-0.116	0.560
	Pf+ Atypical MBC	-0.110	0.583

	Pf+ Naive B cells	0.892	<0.001
	Pf+ Plasma cells/blasts	-0.588	<0.001
	Total frequency of Pf+ B cells	-0.397	0.001
	Schizont-spec IgG	-0.092	0.685
	Schizont-spec IgM	-0.023	0.945
	AMA1	0.012	0.972
	MSP2	0.036	0.920
	CSP	0.170	0.522
non-IgG MBC	Atypical MBC	0.040	0.877
	Naive B cells	-0.233	0.113
	Plasma cells/blasts	-0.014	0.957
	Total frequency of B cells	-0.158	0.379
	Pf+ IgG MBC	0.759	<0.001
	Pf+ non-IgG MBC	0.879	<0.001
	Pf+ Atypical MBC	-0.073	0.783
	Pf+ Naive B cells	-0.436	<0.001
	Pf+ Plasma cells/blasts	-0.034	0.894
	Total frequency of Pf+ B cells	0.394	0.001
	Schizont-spec IgG	0.034	0.897
	Schizont-spec IgM	0.349	0.029
	AMA1	-0.053	0.877
	MSP2	-0.140	0.628
	CSP	-0.021	0.950
Pf+ Atypical MBC	Pf+ Naive B cells	-0.013	0.960
	Pf+ Plasma cells/blasts	-0.344	0.005
	Total frequency of Pf+ B cells	-0.124	0.522
	Schizont-spec IgG	-0.171	0.316
	Schizont-spec IgM	-0.228	0.252
	AMA1	-0.402	0.020
	MSP2	-0.173	0.513
	CSP	0.057	0.872
Pf+ IgG MBC	Pf+ non-IgG MBC	0.700	<0.001
	Pf+ Atypical MBC	0.199	0.191
	Pf+ Naive B cells	-0.469	<0.001
	Pf+ Plasma cells/blasts	-0.081	0.722
	Total frequency of Pf+ B cells	0.365	0.002
	Schizont-spec IgG	0.095	0.673
	Schizont-spec IgM	0.201	0.328
	AMA1	-0.232	0.296
	MSP2	-0.209	0.377
	CSP	-0.095	0.783
Pf+ Naive B cells	Pf+ Plasma cells/blasts	-0.536	<0.001

	Total frequency of Pf+ B cells	-0.416	<0.001
	Schizont-spec IgG	-0.044	0.870
	Schizont-spec IgM	-0.100	0.722
	AMA1	-0.088	0.790
	MSP2	-0.123	0.678
	CSP	0.096	0.783
Pf+ non-IgG MBC	Pf+ Atypical MBC	-0.127	0.516
	Pf+ Naive B cells	-0.418	<0.001
	Pf+ Plasma cells/blasts	-0.030	0.898
	Total frequency of Pf+ B cells	0.226	0.113
	Schizont-spec IgG	0.030	0.905
	Schizont-spec IgM	0.312	0.065
	AMA1	0.085	0.806
	MSP2	-0.043	0.897
	CSP	0.059	0.870
Pf+ Plasma cells/blasts	Total frequency of Pf+ B cells	0.295	0.022
	Schizont-spec IgG	0.101	0.656
	Schizont-spec IgM	-0.020	0.950
	AMA1	0.119	0.700
	MSP2	0.196	0.420
	CSP	-0.288	0.152
Plasma cells/blasts	Total frequency of B cells	0.613	<0.001
	Pf+ IgG MBC	-0.044	0.869
	Pf+ non-IgG MBC	-0.041	0.873
	Pf+ Atypical MBC	-0.312	0.014
	Pf+ Naive B cells	-0.560	<0.001
	Pf+ Plasma cells/blasts	0.971	<0.001
	Total frequency of Pf+ B cells	0.367	0.002
	Schizont-spec IgG	0.112	0.589
	Schizont-spec IgM	-0.046	0.877
	AMA1	-0.015	0.967
	MSP2	0.117	0.710
	CSP	-0.312	0.108
Schizont-spec IgG	Schizont-spec IgM	0.112	0.673
	AMA1	0.070	0.829
	MSP2	0.206	0.376
	CSP	-0.308	0.102
Schizont-spec IgM	AMA1	-0.144	0.666
	MSP2	0.082	0.823
	CSP	-0.064	0.872
Total frequency of B cells	Pf+ IgG MBC	-0.142	0.427
	Pf+ non-IgG MBC	-0.151	0.387

	Pf+ Atypical MBC	-0.223	0.119
	Pf+ Naive B cells	-0.287	0.027
	Pf+ Plasma cells/blasts	0.559	<0.001
	Total frequency of Pf+ B cells	0.166	0.312
	Schizont-spec IgG	0.154	0.397
	Schizont-spec IgM	0.118	0.660
	AMA1	-0.025	0.945
	MSP2	-0.142	0.625
	CSP	-0.183	0.479
Total frequency of Pf+ B cells	Schizont-spec IgG	0.130	0.520
	Schizont-spec IgM	0.129	0.617
	AMA1	-0.203	0.400
	MSP2	-0.149	0.589
	CSP	-0.130	0.667

*Adjusted with false discovery rate (FDR).
