# Supplementary material for: Development of high affinity antibodies to Plasmodium falciparum merozoite and sporozoite antigens during infancy and adulthood
Source: Front Immunol. 2025 Jul 2;16:1562671. doi: 10.3389/fimmu.2025.1562671 (PMC12263619; doi:10.3389/fimmu.2025.1562671)

**Supplementary figure 1**

*Comparison of antibody dissociation rate constants (k_d_) for infants and mothers.* Bars represent median of antibody affinity (k_d_) against MSP2 (A), AMa1 (B) and CSP (C). Error bars represent interquartile range (IQR), and individual values are shown as dots. **** significant at P < 0.0001 tested by Kruskal-Wallis test.

**A**


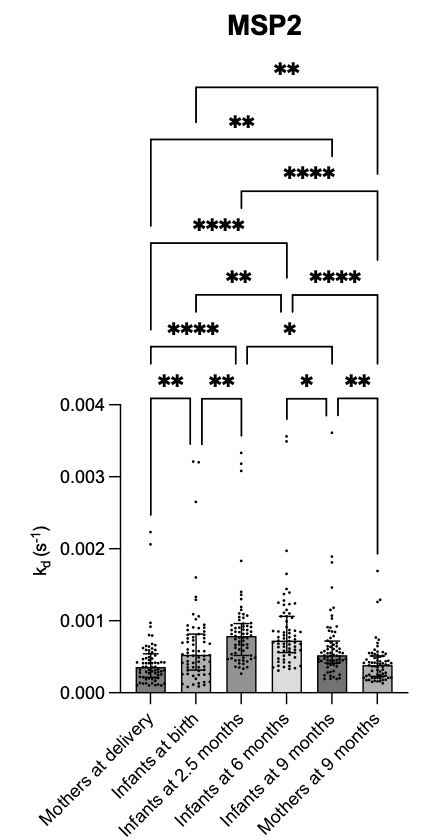


**B C**


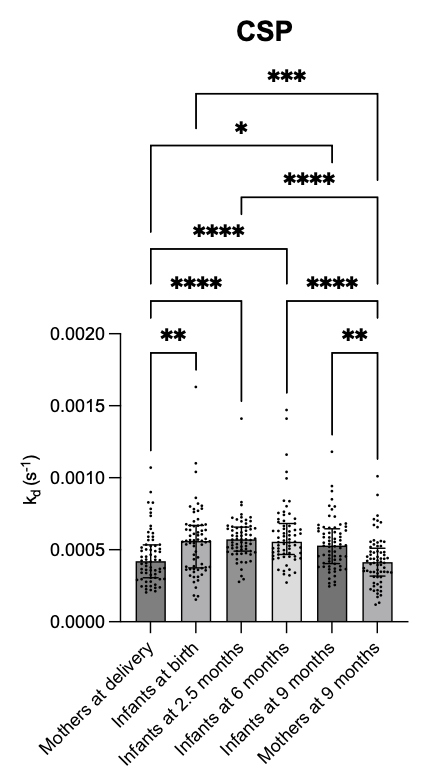

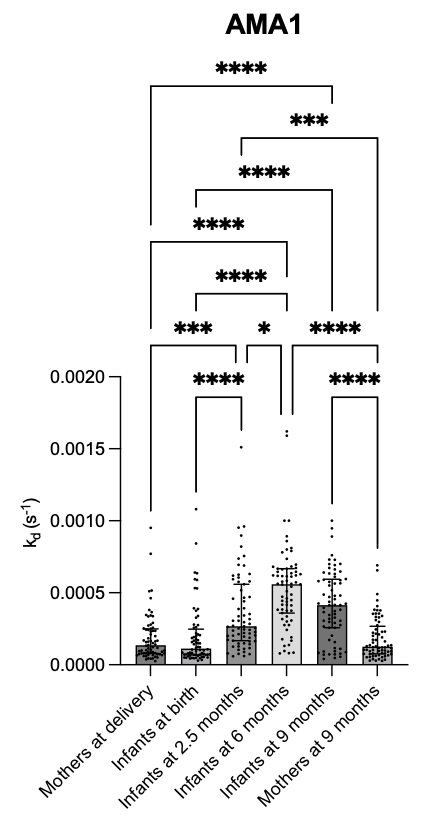

Supplement: Supplementary file 3 [file DataSheet3.docx]
